# Phosphoproteomic and Metabolomic Profiling Uncovers the Roles of CcPmk1 in the Pathogenicity of *Cytospora chrysosperma*

**DOI:** 10.1128/spectrum.00176-22

**Published:** 2022-06-23

**Authors:** Lu Yu, Yuchen Yang, Dianguang Xiong, Chengming Tian

**Affiliations:** a The Key Laboratory for Silviculture and Conservation of Ministry of Education, College of Forestry, Beijing Forestry University, Beijing, China; b Beijing Key Laboratory for Forest Pest Control, Beijing Forestry University, Beijing, China; Université Côte d'Azur, CNRS, Inserm

**Keywords:** CcPmk1, *Cytospora chrysosperma*, CcSte12, secondary metabolism gene cluster, pathogenicity

## Abstract

Pmk1, a highly conserved pathogenicity-related mitogen-activated protein kinase (MAPK) in pathogenic fungi, is phosphorylated and activated by MAP2K and acts as a global regulator of fungal infection and invasive growth by modulating downstream targets. However, the hierarchical CcPmk1 regulatory network in *Cytospora chrysosperma*, the main causal agent of canker disease in many woody plant species, is still unclear. In this study, we analyzed and compared the phosphoproteomes and metabolomes of Δ*CcPmk1* and wild-type strains and identified pathogenicity-related downstream targets of CcPmk1. We found that CcPmk1 could interact with the downstream homeobox transcription factor CcSte12 and affect its phosphorylation. In addition, the Δ*CcSte12* displayed defective phenotypes that were similar to yet not identical to that of the Δ*CcPmk1* and included significantly reduced fungal growth, conidiation, and virulence. Remarkably, CcPmk1 could phosphorylate proteins translated from a putative secondary metabolism-related gene cluster, which is specific to *C. chrysosperma*, and the phosphorylation of several peptides was completely abolished in the Δ*CcPmk1*. Functional analysis of the core gene (*CcPpns1*) in this gene cluster revealed its essential roles in fungal growth and virulence. Metabolomic analysis showed that amino acid metabolism and biosynthesis of secondary metabolites, lipids, and lipid-like molecules significantly differed between wild type and Δ*CcPmk1*. Importantly, most of the annotated lipids and lipid-like molecules were significantly downregulated in the Δ*CcPmk1* compared to the wild type. Collectively, these findings suggest that CcPmk1 may regulate a small number of downstream master regulators to control fungal growth, conidiation, and virulence in *C. chrysosperma*.

**IMPORTANCE** Understanding the pathogenic mechanisms of plant pathogens is a prerequisite to developing effective disease-control methods. The Pmk1 MAPK is highly conserved among phytopathogenic fungi and acts as a global regulator of fungal pathogenicity by modulating downstream transcription factors or other components. However, the regulatory network of CcPmk1 from *C. chrysosperma* remains enigmatic. The present data provide evidence that the core pathogenicity regulator CcPmk1 modulates a few downstream master regulators to control fungal virulence in *C. chrysosperma* through transcription or phosphorylation and that CcPmk1 may be a potential target for disease control.

## INTRODUCTION

Mitogen-activated protein kinase (MAPK) pathways are some of the most important signal transduction pathways that contribute to the response to various external stimuli in eukaryotic organisms (1–3). Generally, MAPK cascades consist of three interlinked protein kinases (PKs), including MAP kinase (MAPK), MAP kinase kinase (MAP2K), and MAP kinase kinase kinase (MAP3K), which are sequentially activated/inactivated through protein phosphorylation/dephosphorylation (3). Subsequently, activated MAPKs phosphorylate downstream targets or directly regulate the transcription of targets such as transcription factors or other components, resulting in corresponding cellular changes. In the model organism budding yeast (Saccharomyces cerevisiae), five classic MAPK pathways have been identified and these pathways are involved in different cellular processes, including the mating and pheromone response pathway (Fus3), filamentous and invasive growth pathway (Kss1), cell integrity pathway (Slt2), high-osmolarity pathway (Hog1), and spore assembly pathway (Smk1) (3). Three well-known MAPK pathways, the Fus3/Kss1 signaling pathway, Hog1 signaling pathway, and Slt2 signaling pathway, are usually found in plant pathogenic fungi. Unlike the numerous MAPK components in plants, ascomycetes contain only a MAPK, MAP2K, and MAP3K in one MAPK signaling pathway (Fus3/Kss1 or Hog1 or Slt2), except Verticillium dahliae and Fusarium oxysporum have two Hog1 orthologs and two Bck1 orthologs, respectively (3–5).

The three different MAPK pathways are involved in convergent and distinct biological functions, such as stress responses, fungal growth, secondary metabolism, and pathogenicity, in plant pathogenic fungi (6–12). Among them, the Fus3/Kss1 MAPK pathway, also referred to as Pmk1 MAPK pathway (pathogenicity-related MAPK), shows highly conserved function in pathogenicity among different plant pathogenic fungi (13–15). Deletion of Pmk1 orthologs in different plant pathogenic fungi leads to reduced virulence or even the loss of pathogenicity (16). For example, *Pmk1* is essential for appressorium formation, penetration, and cell-to-cell extension in Magnaporthe oryzae (6, 17). Similarly, *Pmk1* orthologs are involved in fungal virulence in both appressorium-forming fungi and nonappressorium-forming fungi (7, 18–22). Additionally, *Pmk1* orthologs in Fusarium species are required for the production of mycotoxins, which are important for fungal virulence (23, 24). Similar results have also been found in Aspergillus species (25). Our previous studies showed that *CcPmk1* modulates the expression of gene clusters predicted to be involved in secondary metabolism (26). Interestingly, *Pmk1* orthologs are also found in nonpathogenic fungi and may be responsible for primary metabolism, such as carbohydrate metabolism and protein biosynthesis (25, 27).

The Pmk1 pathway is activated once the fungus perceives the host plant signal or other factors through receptors in the membrane. Subsequently, the signal is transduced intracellularly, resulting in the phosphorylation of Ste11 and sequential activation of Ste7 and Pmk1 through phosphorylation (9). Therefore, phosphorylated Pmk1 regulates the transcription of downstream substrates, such as Ste12 (3). Ste12 is a well-characterized homeodomain transcription factor, and some Ste12 orthologs also contain two tandem C_2_H_2_ zinc fingers at their C terminus (28, 29). Functional analysis of *Ste12* orthologs indicates that they act as core regulators of invasive growth in a variety of phytopathogenic fungi (28, 30–33). In M. oryzae, *mst12* deletion mutants were found to be nonpathogenic on host plants, but they could produce melanized appressoria comparable to those produced by the wild type. Further analyses revealed that the *mst12* deletion mutant could not extend to wound sites, suggesting that *Ste12* is essential for infectious growth (34). However, the *Ste12* ortholog in Sclerotinia sclerotiorum is required for mycelium growth and sclerotia and appressoria formation but not invasive growth (35). Moreover, deletion of *Ste12* orthologs in different phytopathogenic fungi, such as Sordaria macrospora (36), *Cryphonectria parasitica* (37), and Neurospora crassa (38), caused critical defects in sexual spore development, indicating that *Ste12* orthologs are required for mating. Additionally, it has been shown that Ste12 can interact with the MADS-box transcription factor Mcm1 in some plant pathogenic fungi, whose functions in sexual reproduction and pathogenicity seem to overlap (36, 39, 40).

*Cytospora chrysosperma* is a necrotrophic plant pathogenic fungus that can infect over 80 woody plant species, including many economic, shelter, and timber trees, such as poplar, willow, and walnut (41). This fungus causes canker disease on infected trees, and various symptoms are observed on different host species and at different stages of disease development (41). In China, *C. chrysosperma* is the major causal agent of poplar canker disease, which is widespread in poplar planting areas of Northeast, Northwest, and North China. This results in large annual economic and ecological losses. *C. chrysosperma* is an opportunistic pathogen that can penetrate the plant only through wounds and then colonizes the host cells when the plant is under bad conditions. The infected mycelia quickly propagate, disrupt host cells, and spread to adjacent host cells, leading to the deterioration of tissues and death of the branches and twigs. At the late stages of disease development, many obvious black pycnidia are generated on the surface of branches, and orange, sticky conidia are released when it rains. However, the molecular pathogenesis of *C. chrysosperma* remains unknown. Our previous works identified a pathogenicity-related MAPK, *CcPmk1*, which acts as a core component to regulate fungal virulence and development by affecting the expression of downstream genes, including transcription factor genes, effector-encoding genes, kinase genes, and so on (18, 26). For example, the Gti1/PacII transcription factor *CcSge1* and the putative effector *CcCAP1*, both of which are required for fungal virulence, are transcriptionally regulated by *CcPmk1* (42, 43). However, the further mode of action of CcPmk1 is still unclear.

In this study, we performed phosphoproteomic and metabolomic analyses of the Δ*CcPmk1* and wild type. Thousands of phosphorylated peptides were detected in both the Δ*CcPmk1* and wild type. Hundreds of phosphorylated proteins were differentially regulated in the Δ*CcPmk1* compared to the wild type, and these proteins are mainly involved in intracellular hydrolase activity (Gene Ontology [GO]:0016787), protein kinase activity (GO:0004672), and integral component of membrane (GO:0016021), indicating the transformation of survival strategies after deletion of *CcPmk1*. Furthermore, the downstream targets of *CcPmk1*, *CcSte12*, and *CcPpns1* (core gene of a putative secondary metabolism gene cluster) were found to be essential for fungal growth and virulence in *C. chrysosperma*. Metabolomic analysis showed that amino acid metabolism, biosynthesis of secondary metabolites, lipids, and lipid-like molecules significantly changed after the deletion of *CcPmk1*. These results suggest that CcPmk1 acts as a core regulator of fungal pathogenicity through a small number of master downstream targets.

## RESULTS

### Overview of the phosphoproteomic data.

**FIG 1:**
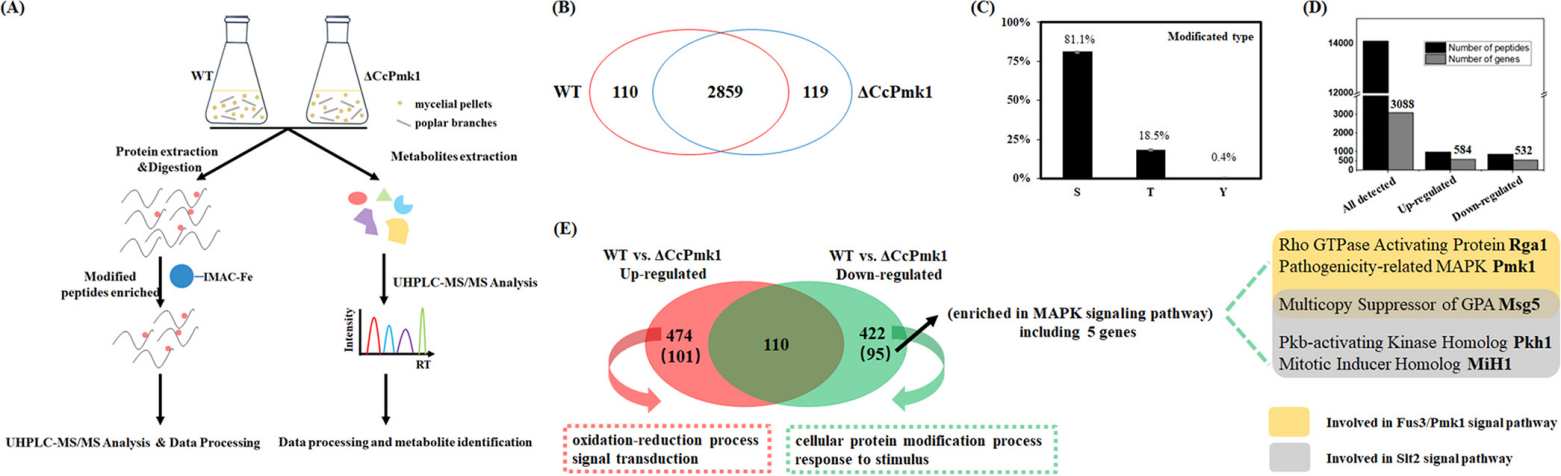
Quantification of phosphoproteomic data between the Δ*CcPmk1* and wild-type strains. (A) Multiomics mass spectrometry-based strategy used to evaluate the molecular profiles of the Δ*CcPmk1* and wild-type strains. (B) Venn diagram showing overlapping phosphorylated proteins with known phosphorylated sites in the Δ*CcPmk1* and wild-type strains. (C) Bar graph showing the distributions of modified Ser, Thr, and Tyr amino acids. (D) Summary of the identified phosphorylated peptides and proteins. The upregulated and downregulated represent peptides and corresponding genes whose abundance was significant different in the Δ*CcPmk1* compared to the wild type. (E) Functional annotation of the significantly regulated phosphosites and corresponding proteins. The numbers in the bracket indicate the phosphorylated proteins contained phosphorylation peptides with no abundance in the wild type or *CcPmk1* deletion mutant. Kyoto Encyclopedia of Genes and Genomes (KEGG) enrichment analysis of the 95 downregulated proteins showed significant enrichment in the MAPK signaling pathway. LC-MS/MS, liquid chromatography–mass spectrometry/mass spectrometr; UHPLC, ultra-high-performance liquid chromatography.

To reveal the changes in phosphorylation events after the deletion of *CcPmk1*, phosphoproteomic analysis was conducted in the wild type and *CcPmk1* deletion mutant during simulated infection. Briefly, the wild-type and Δ*CcPmk1* strains were grown in potato dextrose broth (PDB) medium supplemented with sterilized poplar branches to mimic infection with shaking at 150 rpm and 25°C for 2 days. Then, total protein was extracted and digested from three biological replicates, respectively. Subsequently, the phosphorylated peptides were enriched by using IMAC-Fe, and then the peptides were used for liquid chromatography–mass spectrometry/mass spectrometry (LC-MS/MS) analysis (Fig. 1A). According to phosphoproteomic data, a total of 13,943 and 13,932 phosphorylation peptides were detected in the Δ*CcPmk1* and wild type, respectively. After phosphorylated peptides with unidentified sites (the instrument could not distinguish the precise phosphorylation sites in some peptides that contain more than one Ser, Thr, or Tyr residue) were removed, a total of 2,978 and 2,969 phosphorylated proteins were detected in the *CcPmk1* deletion mutant and wild type, respectively (Fig. 1B). Among them, 2,859 proteins were detected in both the *CcPmk1* deletion mutant and wild type, while 110 and 119 phosphorylated proteins were specifically detected in the *CcPmk1* deletion mutant and wild type, respectively (Fig. 1B). Analysis of the phosphorylated amino acids showed that serine residues accounted for the majority of modified residue types (81.1%), while phosphorylated threonine residues (18.5%) and tyrosine residues (0.4%) accounted for a small proportion, especially tyrosine residues (Fig. 1C). The phosphorylation patterns of serine, threonine, and tyrosine residues were similar to the results found in many other organisms (44, 45).

### Functional annotation of the phosphoproteomes.

Based on quantitation of the phosphorylated peptides in the wild type and *CcPmk1* deletion mutant, the peptides whose abundance was significantly altered were analyzed with the criterion of fold change (FC) > 1.5 (upregulated) or FC < 0.67 (downregulated) and *P ≤ *0.05. Here, a total of 981 and 861 peptides (with 331 and 258 peptides in an S/T proline directed motif) were significantly increased/reduced in abundance in the *CcPmk1* deletion mutant compared to the wild type, which corresponded to 584 proteins and 532 proteins, respectively (Fig. 1D). Among them, 110 proteins contained both significantly increased and reduced abundances phosphorylated peptides in the *CcPmk1* deletion mutant compared to the wild type, and 474 proteins contained significantly increased abundances phosphorylated peptides, while 422 proteins contained significantly reduced abundances phosphorylated peptides (Fig. 1E). In addition, we found that the abundance of 151 phosphorylated peptides was abolished in Δ*CcPmk1*, and among them, 51 phosphorylated peptides contained the MAPK phosphorylation motif S/T-P. Gene Ontology (GO) analysis showed that the cellular protein modification process and response to stimulus were annotated in the 422 proteins that contained significantly reduced phosphorylated proteins in the *CcPmk1* deletion mutant compared to the wild type, while the oxidation-reduction process and signal transduction were annotated in the 474 proteins that contained significantly increased phosphorylated proteins in the *CcPmk1* deletion mutant compared to the wild type (Fig. 1E). Additionally, Kyoto Encyclopedia of Genes and Genomes (KEGG) enrichment analysis of 95 proteins, which contained phosphorylation peptides with no abundance in Δ*CcPmk1*, showed that the MAPK signaling pathway was significantly enriched, including 5 proteins, Rho GTPase activating protein Rga ortholog (GME1269_g), pathogenicity-related MAPK Pmk1 ortholog (CcPmk1), multicopy suppressor of GPA Msg5 ortholog (GME6737_g), Pkb-activating kinase homolog Pkh1 (GME8781_g), and mitotic inducer homolog MiH1 (GME5846_g), which are involved in the Fus3/Pmk1 signaling pathway and/or Slt2 signaling pathway (Fig. 1E).

**FIG 2:**
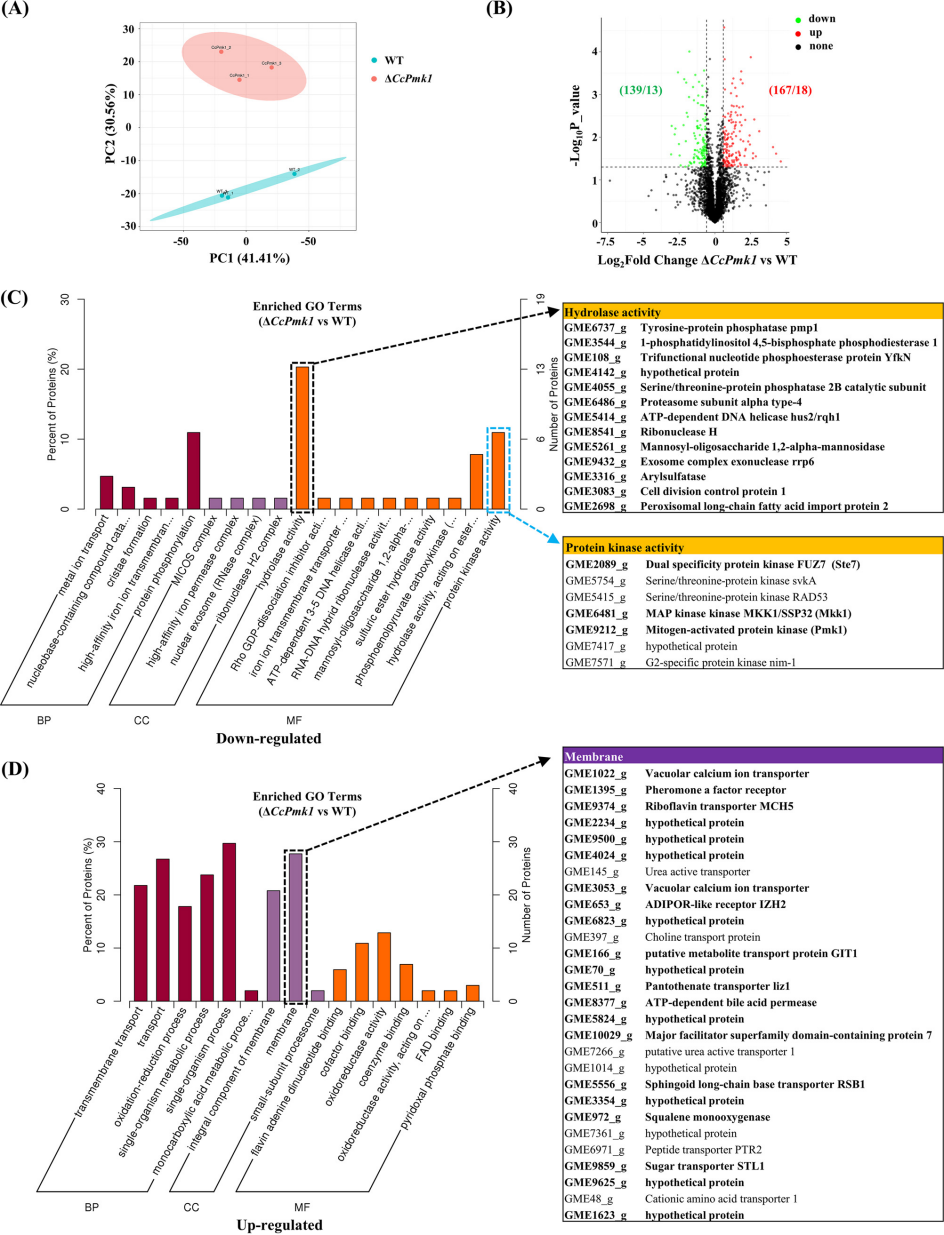
Functional annotation of significantly regulated phosphoproteins. (A) principal-component analysis (PCA) of the Δ*CcPmk1* and wild-type samples. (B) The volcano diagram shows the phosphoproteins whose abundance was significant different in the Δ*CcPmk1* compared to the wild type. The significantly expressed proteins were defined based on *P < *0.05 and (fold change >1.5 or <0.67). (C) Significantly enriched Gene Ontology (GO) terms of differentially downregulated proteins in the cellular component, molecular function, and biological process categories. The proteins in bold are involved in the phosphatase or MAPK signaling pathway. (D) Significantly enriched GO terms of differentially upregulated proteins in the cellular component, molecular function, and biological process categories. The proteins in bold are involved in the integral component of membrane. BP, biological process; CC, cellular component; MF, molecular function.

To reveal the changes in phosphorylated protein levels, the abundance of phosphorylated proteins was calculated and normalized using all detected phosphorylated peptides. Then, we evaluated the relationship between three replicates each of the *CcPmk1* deletion mutant and the wild type by using the principal-component analysis (PCA), and the results showed that the three replicates each of the *CcPmk1* deletion mutant and wild type clustered together. Additionally, the *CcPmk1* deletion mutant samples and wild-type samples were obviously separated (Fig. 2A), indicating the reliability of the sampling and results. Subsequently, the abundance of 306 phosphorylated proteins significantly differed in the *CcPmk1* deletion mutant compared to the wild type, including 139 proteins with significantly increased abundance and 167 proteins with significantly reduced abundances (Fig. 2B). Among them, the abundance of 13 phosphorylated proteins, including CcPmk1 itself, was completely abolished in the *CcPmk1* deletion mutant, but these phosphorylated proteins were detected in the wild type (Fig. 2B). Importantly, four of these phosphorylated proteins (GME3439_g, GME5111_g, GME5414_g, GME9931_g) contain the typical MAPK phosphorylation motif S/T-P, which might be the directly phosphorylated target of CcPmk1. Functional annotation of these 12 proteins (except CcPmk1) revealed that 8 out of the 12 were annotated as hypothetical proteins (Table 1). Remarkably, GME3434_g and GME3439_g, annotated as oxidereductase and 1-aminocyclopropane-1-carboxylate oxidase, respectively, were predicted to be translated from genes related to secondary metabolism in our previous study, and these genes are also transcriptionally regulated by CcPmk1 (26).

**TABLE 1:** Phosphorylated proteins completely abolished their abundances in the *CcPmk1* deletion mutant

Protein ID	Protein description	GO_description^*a*^
GME3434_g^*b*^	Oxidereductase, NAD binding domain cpaG	Molecular function: oxidoreductase activity (GO:0016491)
GME3439_g^*b*^^,^^*c*^	1-Aminocyclopropane-1-carboxylate oxidase	Molecular function: oxidoreductase activity (GO:0016491) Biological process: oxidation-reduction process (GO:0055114)
GME5111_g^*c*^	Hypothetical protein	Biological process: cristae formation (GO:0042407) Cellular component: MICOS complex (GO:0061617)
GME9212_g (CcPmk1)	Mitogen-activated protein kinase	Molecular function: protein kinase activity (GO:0004672) Molecular function: ATP binding (GO:0005524), Biological process: protein phosphorylation (GO:0006468)
GME5414_g^*c*^	ATP-dependent DNA helicase hus2/rqh1	Molecular function: nucleic acid binding (GO:0003676), ATP binding (GO:0005524), ATP-dependent 3′–5′ DNA helicase activity (GO:0043140) Biological process: DNA replication (GO:0006260), DNA repair (GO:0006281)
GME9931_g^*c*^	Hypothetical protein	
GME8328_g	Hypothetical protein	
GME7802_g	Hypothetical protein	
GME368_g	Importin subunit beta-5	Biological process: intracellular protein transport (GO:0006886) Molecular function: Ran GTPase binding (GO:0008536)
GME2524_g	Hypothetical protein	
GME9679_g	Hypothetical protein	Molecular function: methyltransferase activity (GO:0008168) Biological process: methylation (GO:0032259)
GME9494_g	Hypothetical protein	
GME428_g	Hypothetical protein	

a GO, Gene Ontology.

b Putative secondary metabolism involving proteins.

c Phosphorylated peptides contained the MAPK phosphorylation motif: S/T-P.

GO analysis of the proteins whose phosphorylation was significantly changed showed that protein phosphorylation (GO:0006468), hydrolase activity (GO:0016787), and PK activity (GO:0004672) were significantly enriched in the downregulated proteins in the *CcPmk1* deletion mutant compared to the wild type (Fig. 2C). The enriched proteins involved in hydrolase activity are mainly associated with intracellular hydrolase activity, and four of them (GME6763_g, GME3544_g, GME108_g, and GME4055_g) are phosphatases. Additionally, the enriched proteins Ste7 ortholog (GME2089_g), Mkk1 ortholog (GME6481_g), and Pmk1 ortholog (GME9212_g) exhibit PK activity (Fig. 2C). GME2089_g and GME6481_g are MAP2Ks of the Pmk1 signaling pathway and Slt2 signaling pathway, respectively, indicating feedback regulation of MAPK signaling pathways and putative cross talk between the Pmk1 signaling pathway and Slt2 signaling pathway (Fig. 2C). On the other hand, the GO terms enriched in the significantly upregulated proteins were transmembrane transport (GO:0055085), oxidation-reduction process (GO:0055114), single-organism process (GO:0044710:), membrane (GO:0016020), and oxidoreductase activity (GO:0016491) (Fig. 2D). As for membrane-related terms, over 20 proteins are involved in integral component of membrane (GO:0016021). These results suggest that *C. chrysosperma* may have transformed its living strategies to overcome defects after the deletion of *CcPmk1*.

### Analysis of changes in the phosphorylation of PKs and transcription factors.

PKs are involved in various biological processes and respond to environmental changes through reversible protein phosphorylation. In eukaryotic organisms, different PKs, such as the AGC, STE, CMGC, TKL, CAMK, RGC, CK1, and atypical PKs, have been identified based on sequence similarity, domain structures, and regulation patterns (46). Here, we systemically identified PKs in the *C. chrysosperma* genome, and a total of 128 PK genes were obtained (data not shown). Among them, the abundance of nine PKs was significantly different in the *CcPmk1* deletion mutant compared to the wild type, including seven downregulated PKs and two upregulated PKs (Table S2). Homolog analyses of these downregulated PKs in the pathogen-host interaction database (http://www.phi-base.org/) showed that these PKs orthologs have been systematically and functionally characterized in Fusarium graminearum (24). Among the nine differentially regulated PKs, four of their orthologs in F. graminearum are required for fungal virulence including the three MAPKs orthologs MKK1, FUS3, and STE7, while one out of seven is dispensable for fungal pathogenicity, and two of them have not been functionally identified (Table S2), suggesting that CcPmk1-associated PKs may play important roles in fungal pathogenicity.

**FIG 3:**
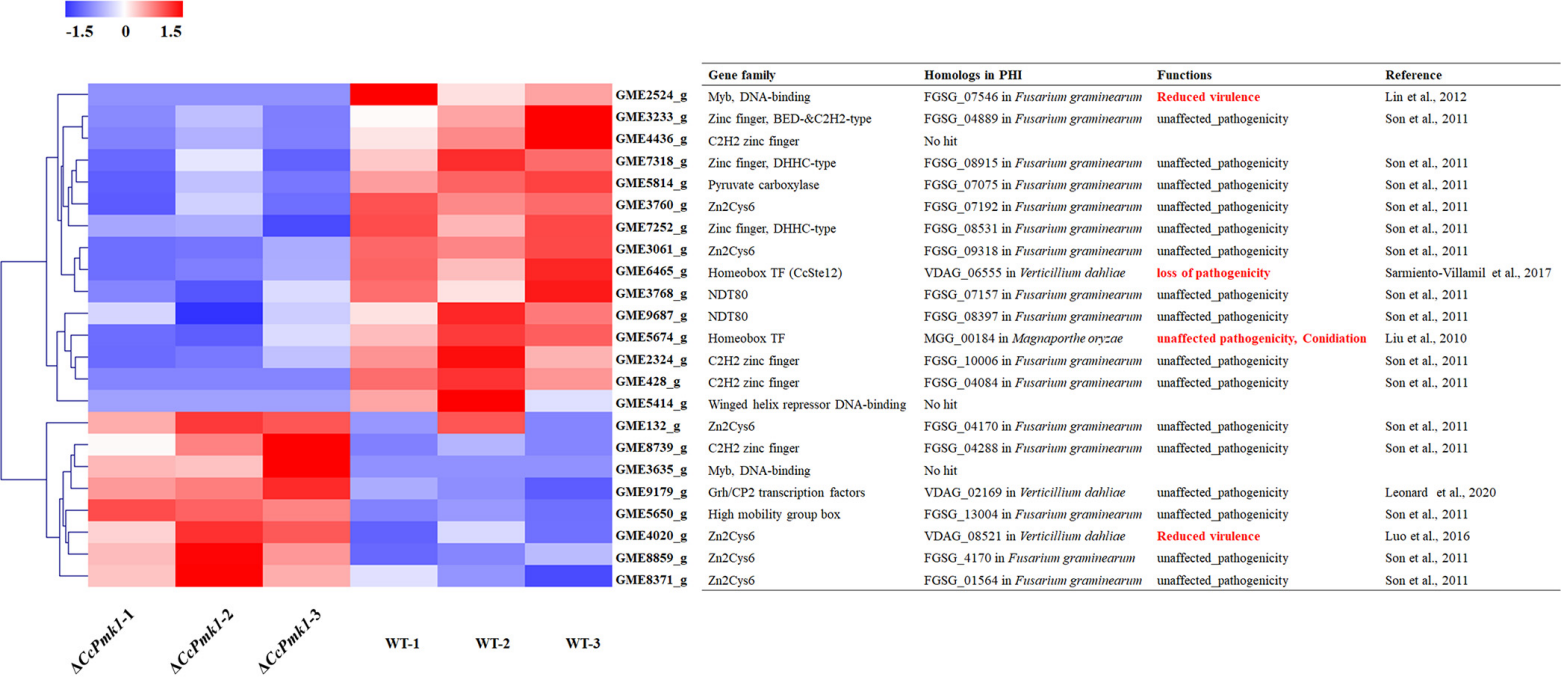
Heatmap showing the temporal abundance of transcription factors whose abundance differed in the Δ*CcPmk1* compared to the wild type. Levels represent the normalized abundance of the phosphorylated proteins. The homologs of these transcription factors that are essential for fungal pathogenicity and conidiation are labeled in red font (48, 58, 63–66). PHI, Pathogen Host Interactions database.

Additionally, we calculated the putative downstream transcription factors whose phosphorylation was regulated by CcPmk1. Then, we calculated a total of 23 transcription factors whose protein abundance was significantly different in the *CcPmk1* deletion mutant compared to the wild type, including 15 downregulated transcription factors and 8 upregulated transcription factors, which were annotated as Zn_2_Cys_6_, C_2_H_2_, DHHC-type zinc finger, homeobox/homeodomain, Winged helix repressor DNA-binding, and so on (Fig. 3). Among them, the homeobox transcription factor Ste12 ortholog (GME6465_g, named CcSte12 in this study) showed significantly reduced phosphorylated levels in the *CcPmk1* deletion mutant, which is one of the well-characterized downstream targets of Pmk1 and is indispensable for invasion growth in many fungi species. Furthermore, only two of the homologs (GME2524_g, Myb DNA-binding and GME4020_g, Zn_2_Cys_6_) were required for fungal virulence, in addition to the CcSte12 homolog (Fig. 3). Additionally, the GME5674_g (a homeobox transcription factor) homolog MGG_00184 is essential for conidiogenesis in M. oryzae (47, 48). These results suggest that CcPmk1 may regulate fungal virulence via a small number of master downstream transcription factors.

### CcSte12 is phosphorylated by CcPmk1 and is required for fungal pathogenicity.

**FIG 4:**
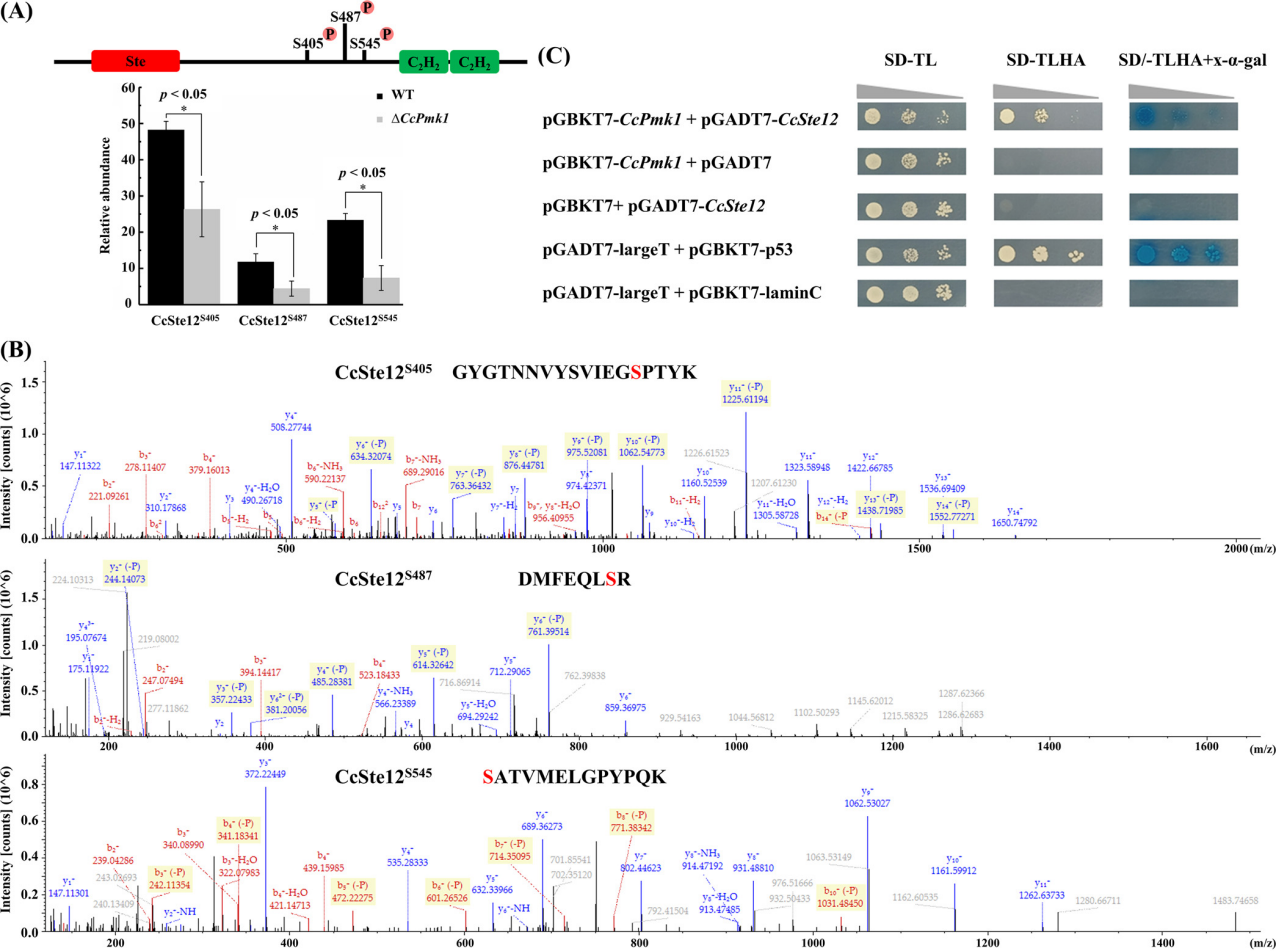
Phosphorylation of the homeobox transcription factor CcSte12. (A) Significantly reduced abundance of phosphorylation sites of CcSte12 in the Δ*CcPmk1* compared to the wild type. (B) The three phosphorylated residues Ser405, Ser487, and Ser545 in CcSte12 were identified by mass spectrometry. (C) Yeast two-hybrid assay between CcPmk1 and CcSte12. SD-TL refers to SD medium lacing Trp and Leu. SD-TLHA refers to SD medium lacing Trp, Leu, His, and Ade.

As described above, the phosphorylation level of the homeobox transcription factor CcSte12 (GME6465_g), which is a well-known transcription factor downstream of Pmk1 (29), was significantly reduced in the *CcPmk1* deletion mutant compared to the wild type. Therefore, we analyzed the phosphorylated residues in CcSte12. As shown in Fig. 4A, the abundance of peptides that included the phosphorylated residues Ser405, Ser487, and Ser545 was significantly reduced in the *CcPmk1* deletion mutant compared to the wild type. Moreover, the phosphorylated residue Ser405 harbored a MAPK S/T-P phosphorylation motif. The mass spectra showing these phosphorylated residues (Ser405, Ser487, and Ser545) are shown in Fig. 4B. Additionally, we determined the interaction between CcPmk1 and CcSte12 by using yeast two-hybrid assays. The results clearly showed that CcPmk1 could interact with CcSte12 (Fig. 4C).

**FIG 5:**
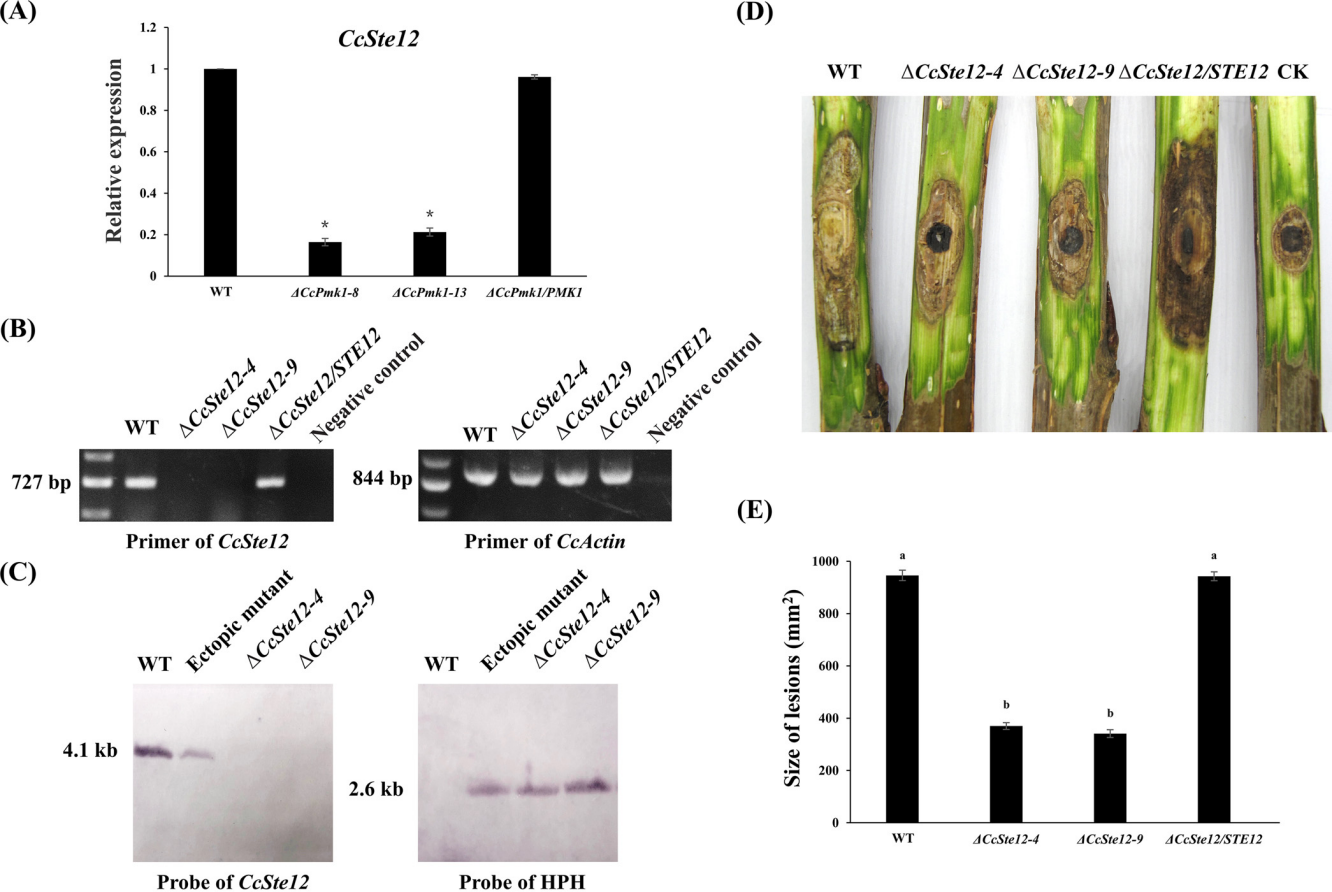
*CcSte12* is transcriptionally regulated by *CcPmk1* and required for fungal virulence. (A) The expression levels of *CcSte12* in Δ*CcPmk1* mutant. The asterisks indicate significant differences at *P < *0.05. (B) The Δ*CcSte12* and the complemented strain were screened by using PCR amplification with specific internal and *Ccactin* primer pairs. Negative control indicates that no template was used. (C) Southern blot analysis of genomic DNA from the wild type and two deletion mutants. Genomic DNA from these isolates was digested with XhoI. The enzyme-digested products were probed with a DNA sequence from the hph gene and the sequence of *CcSte12*. (D) The pictures show poplar twigs inoculated with the wild-type, Δ*CcSte12* mutants, and complemented strains. CK refers to twigs inoculated with 5-mm agar plugs of potato dextrose agar (PDA). (E) The statistical data for lesion areas on the poplar twigs inoculated with each strain. ^a,b^*P < *0.05, different letters indicate significant differences. Error bars represent SD based on three independent biological replicates with three technical replicates each. The data were analyzed using one-way ANOVA followed by Duncan’s range test with SPSS 20.0.

**FIG 6:**
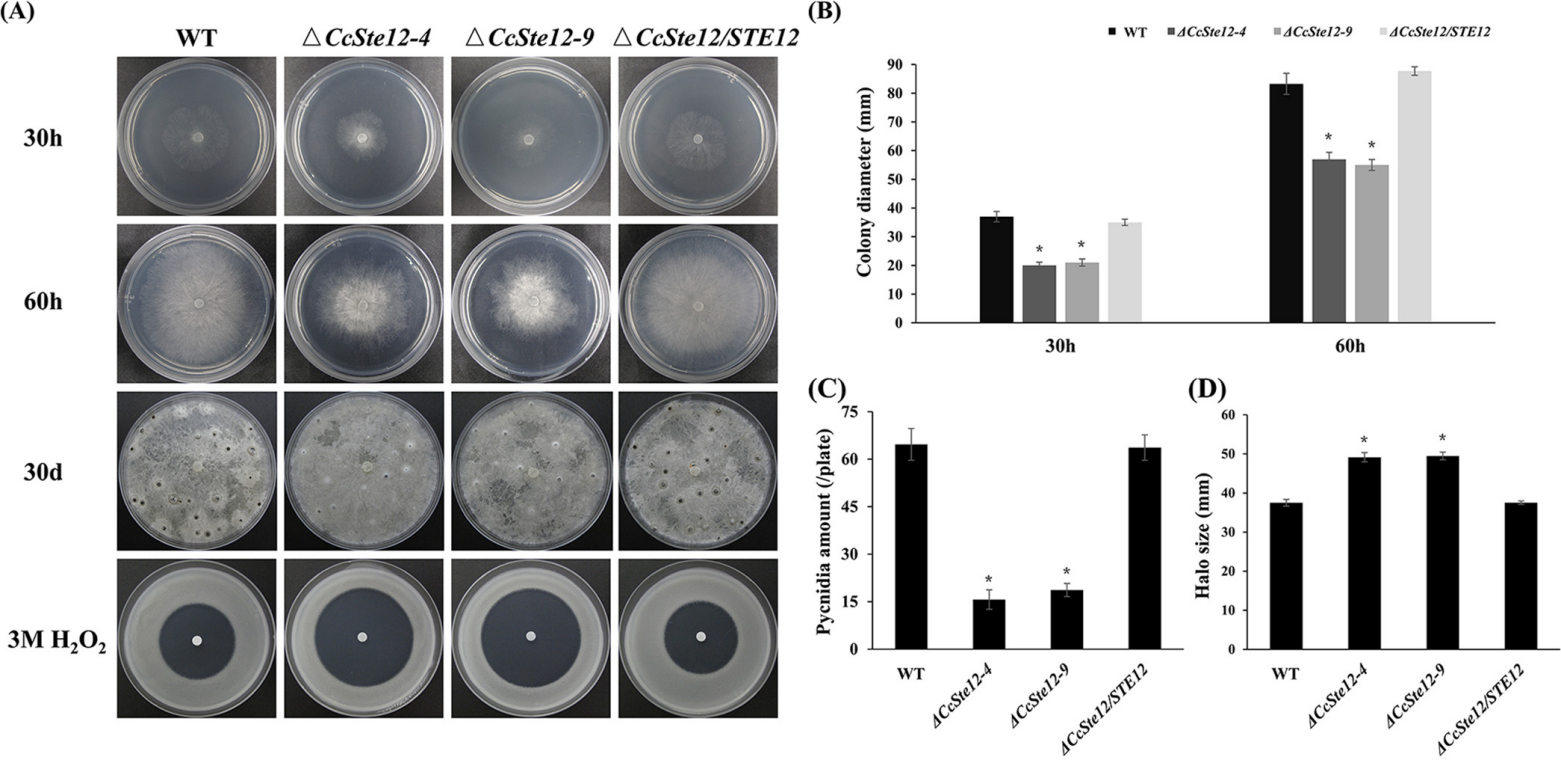
CcSte12 is involved in fungal growth, conidiation, and stress responses. (A) Colony morphology of the wild-type, Δ*CcSte12*, and complemented strains upon incubation on plates containing PDA or PDA supplemented with 3 M H_2_O_2_. (B) Colony diameter of each strain after 30 h and 60 h of growth on PDA plates. The values show the average of three colony diameter measurements. (C) Average number of pycnidia after 30 days of growth on PDA medium based on three independent experiments. (D) Average halo diameters after 3 days of growth on PDA medium supplemented with 3 M H_2_O_2_ based on three independent experiments. Error bars represent SD based on three independent biological replicates with three technical replicates each. The data were analyzed using one-way ANOVA followed by Duncan’s range test with SPSS 20.0. *, *P < *0.05, significant differences.

Subsequently, we calculated the expression levels of *CcSte12* in the *CcPmk1* deletion mutant and found it to be significantly reduced, indicating that *CcSte12* is also transcriptionally regulated by *CcPmk1* (Fig. 5A). Then, we deleted *CcSte12* in *C. chrysosperma* by using the split-marker method, and two single-copy replacement mutants were successfully acquired according to PCR confirmation and Southern blot analysis (Fig. 5B and C). As expected, the virulence of the *CcSte12* deletion mutant was significantly compromised compared to that of the wild-type and complemented strains (Fig. 5D and E). Phenotypic analyses revealed that deletion of *CcSte12* also caused obvious defects in fungal growth, conidiation, and sensitivity to hydrogen peroxide. The Δ*CcSte12* deletion mutant showed an ~35% reduction in radial growth, an ~75% reduction in conidiation, and greater sensitivity to hydrogen peroxide than the wild-type and complemented strains (Fig. 6). Comparing the phenotypes of the Δ*CcSte12* and Δ*CcPmk1* mutant, we found that they shared many similar defects in fungal growth, conidiation, and fungal pathogenicity. However, defects in the *CcPmk1* deletion mutant, such as loss of pathogenicity and no conidiation, were more serious than those in the *CcSte12* deletion mutant (18). Taken together, these results showed that the functions of CcPmk1 can be partly achieved by the downstream transcription factor CcSte12 through many mechanisms, including transcriptional regulation, phosphorylation, and complex formation.

Recently, Osés-Ruiz et al. (22) found that a pathogenicity-related homeobox transcription factor named MoHox7, which acts as a downstream target of MoPmk1 in M. oryzae, is transcriptionally regulated and phosphorylated by MoPmk1. Therefore, we identified the *MoHox7* ortholog in *C. chrysosperma* and named it *CcHox7* (*GME6368_g*). We found that CcHox7 could be phosphorylated at residues Thr60, Ser153, and Ser172 (Fig. S1). Remarkably, CcHox7^S153^ and CcHox7^S172^ contain a typical MAPK S/T-P phosphorylation motif. However, the modified proteins abundance of CcHox7 did not significantly differ in the *CcPmk1* deletion mutant compared to the wild type (Table S3). Additionally, the phosphorylation sites in MoHox7 and CcHox7 were not conserved, although the sequences share high identity (Fig. S1). Additionally, CcPmk1 could weakly interact with CcHox7 according to yeast two-hybrid assays (Fig. S2). Furthermore, we systematically identified the homeobox or homeodomain transcription factors in the *C. chrysosperma* genome and then determined whether their phosphorylation is regulated by CcPmk1. However, only two of the seven homeobox or homeodomain transcription factors (CcSte12 and GME5674_g) were significantly reduced in abundance in the *CcPmk1* deletion mutant compared to the wild type, and the abundance of one modified peptide from GME5631_g was significantly reduced (Table S3). In addition, GME5631_g^S176^, GME5631_g^T485^, GME5674_g^S625^, GME5674_g^S637^, and CcSte12^S314^ contain a MAPK phosphorylation S/T-P motif, even if the abundance of these modified peptides was not significantly changed in the *CcPmk1* deletion mutant. The results indicated that CcPmk1 may regulate the functions of homeobox transcription factors in addition to CcSte12.

### The putative secondary metabolism-related proteins were phosphorylated by CcPmk1.

**FIG 7:**
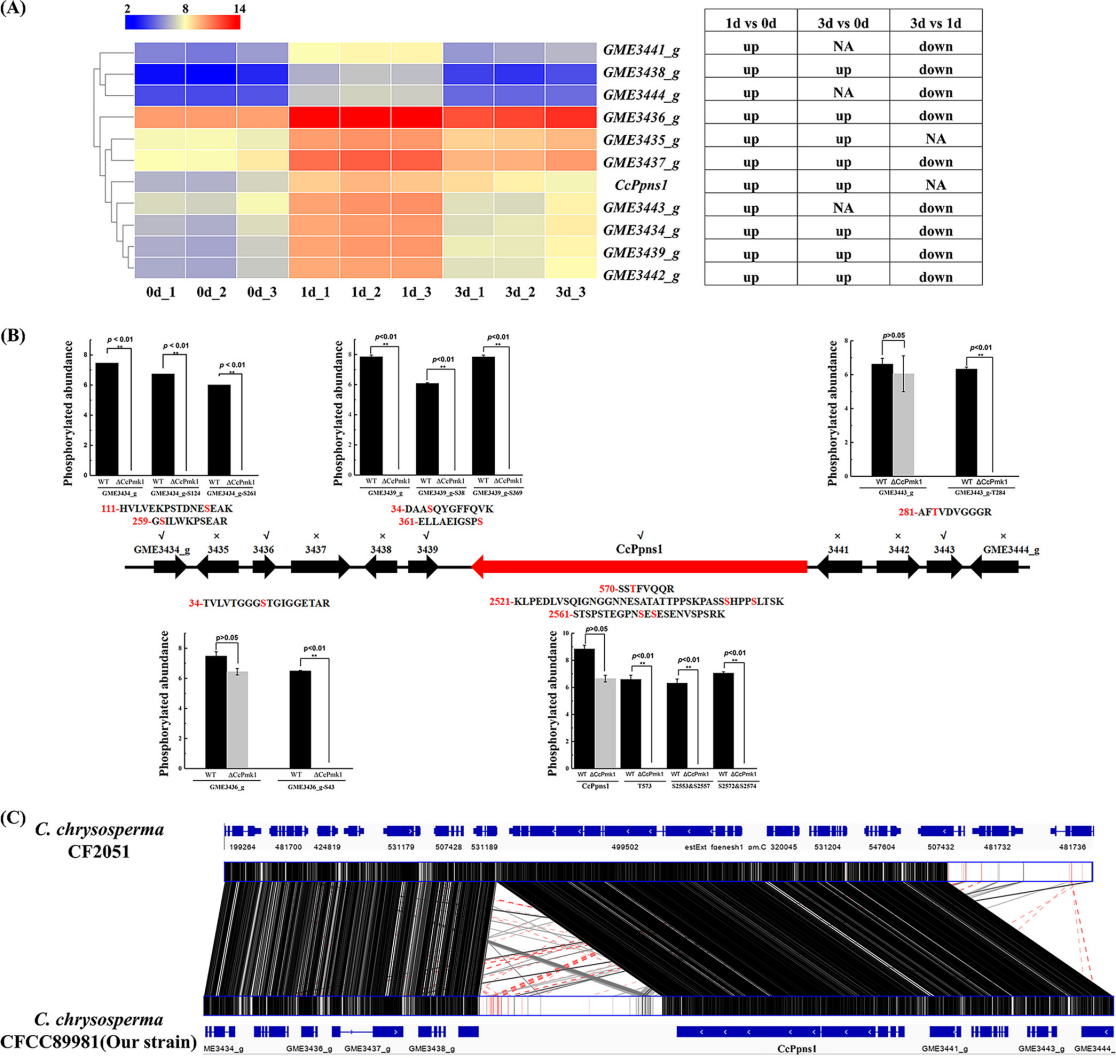
Phosphorylation of a putative secondary metabolism gene cluster (*GME3434_g* to *GME3444_g*). (A) The heatmap shows the expression patterns of the genes in the cluster at the initial stages of infection. The original fragments per kilobase per million values of the cluster genes were log_2_ transformed. The color scale ranging from blue to red indicates increasing expression levels. Differentially expressed genes (|log2fold change| ≥ 1, *P < *0.05) are indicated. The “up” indicates significant upregulation, while the “down” indicates significant downregulation. (B) The transcript arrangement and phosphorylated protein abundance of the cluster. The line with the arrow indicates the transcript arrangement of the gene cluster. The bar chart shows the abundance of phosphorylated proteins and phosphorylated residues in the *CcPmk1* deletion mutant compared to the wild type. The “√” indicates that the phosphorylation of these proteins was detected in the Δ*CcPmk1* and wild type. The “×” indicates that no phosphorylation of these proteins was detected in the Δ*CcPmk1* and wild type. **, *P < *0.01, significant differences. The red residues in the peptide sequences represent phosphorylation sites. The red gene box indicates the core gene in the gene cluster. (C) Global view of syntenic alignments of secondary metabolism gene clusters in two *C. chrysosperma* strains. The gene arrangement was visualized by IGV with gene annotation files downloaded from each genome database. The lines connecting the two boxes are colored black to indicate +/+ oriented alignment.

As shown in Table 1, the phosphorylation of the secondary metabolism-related proteins GME3434_g and GME3439_g was completely abolished in the Δ*CcPmk1*. Our previous work found that CcPmk1 regulates expression of the whole gene cluster from *GME3434_g* to *GME3444_g*. Here, we collected expression data for these cluster genes from our recent transcriptome data during the initial *C. chrysosperma* infection process (1 and 3 days postinfection [dpi]) of poplar branches (49). As shown in Fig. 7A, the expression levels of all cluster genes were significantly upregulated at 1 dpi compared to 0 dpi, and most of the genes (8/11) were also significantly upregulated at 3 dpi compared to 0 dpi. However, 9 out of 11 genes were significantly downregulated at 3 dpi compared to 1 dpi. These results suggest that this gene cluster may play important roles in the fungal pathogenicity of *C. chrysosperma*. Subsequently, we determined the phosphorylation status of the whole cluster and found that the phosphorylation of 5 out of 11 proteins, GME3434_g, GME3436_g, GME3439_g, GME3440_g (core gene), and GME3443_g, is regulated by CcPmk1 (Fig. 7B). According to the domain structures shown in Fig. S3A, the core gene GME3440_g is a *CcPmk*1-regulated typical polyketide synthase-nonribosomal peptide synthetase (PKS-NRPS), and we named it as *CcPpns1*. Intriguingly, the total phosphorylation levels of *CcPpns1* of the Δ*CcPmk1* and wild type were comparable, but the phosphorylation of *CcPpns1* at positions T573, S2553&S2557, and S2572&S2574 was completely abolished in the Δ*CcPmk1*. Similar results were also found for GME3436_g and GME3443_g. The phosphorylation of GME3436_g at position S43 and the phosphorylation of GME3443_g at position T284 were completely abolished in the Δ*CcPmk1* (Fig. 7B). Domain structure analyses revealed that most of the phosphorylated sites are located in the domain regions (Fig. S3A), which suggests that the phosphorylation of these proteins or peptides by CcPmk1 may be important for their functions. Furthermore, we aimed to determine the putative homologs of genes in this gene cluster in other fungal species. Therefore, all gene sequences in the gene cluster were used as queries to search the genome sequences of M. oryzae, V. dahliae, Botrytis cinerea, *Vala mali* (*Cytospora mali*), Aspergillus species, Fusarium species, and several plant species (poplar, Arabidopsis thaliana, and Nicotiana benthamiana). However, no significant hits were obtained in the fungal species or plant species. To reveal the putative functions of *CcPpns1*, we identified the putative homologs of *CcPpns1* in the NCBI database, and many homologs were found in different fungal species, including an uncharacterized protein in *C. parasitica* (99% sequence coverage with > 75% sequence identity, XP_040777604.1) and lovastatin nonaketide synthases (LNKS) in *Dissoconium aciculare* (99% sequence coverage with > 60% sequence identity, XP_033455183.1). Then, we aligned the sequence of the gene clusters in *C. chrysosperma* and *C. parasitica* and found that only the core gene and several other regions showed high sequence identity (Fig. S3B). Importantly, the whole gene cluster was found in a different strain (CFL2056) of *C. chrysosperma* (https://genome.jgi.doe.gov/Cytch1/Cytch1.home.html), and synteny analysis revealed that the gene clusters in the two *C. chrysosperma* strains showed high sequence identify and a similar gene arrangement, but a fragment insertion (~10 kb) in the gene cluster of our strain was identified (Fig. 7B). These results suggest that this gene cluster is specific to *C. chrysosperma*.

**FIG 8:**
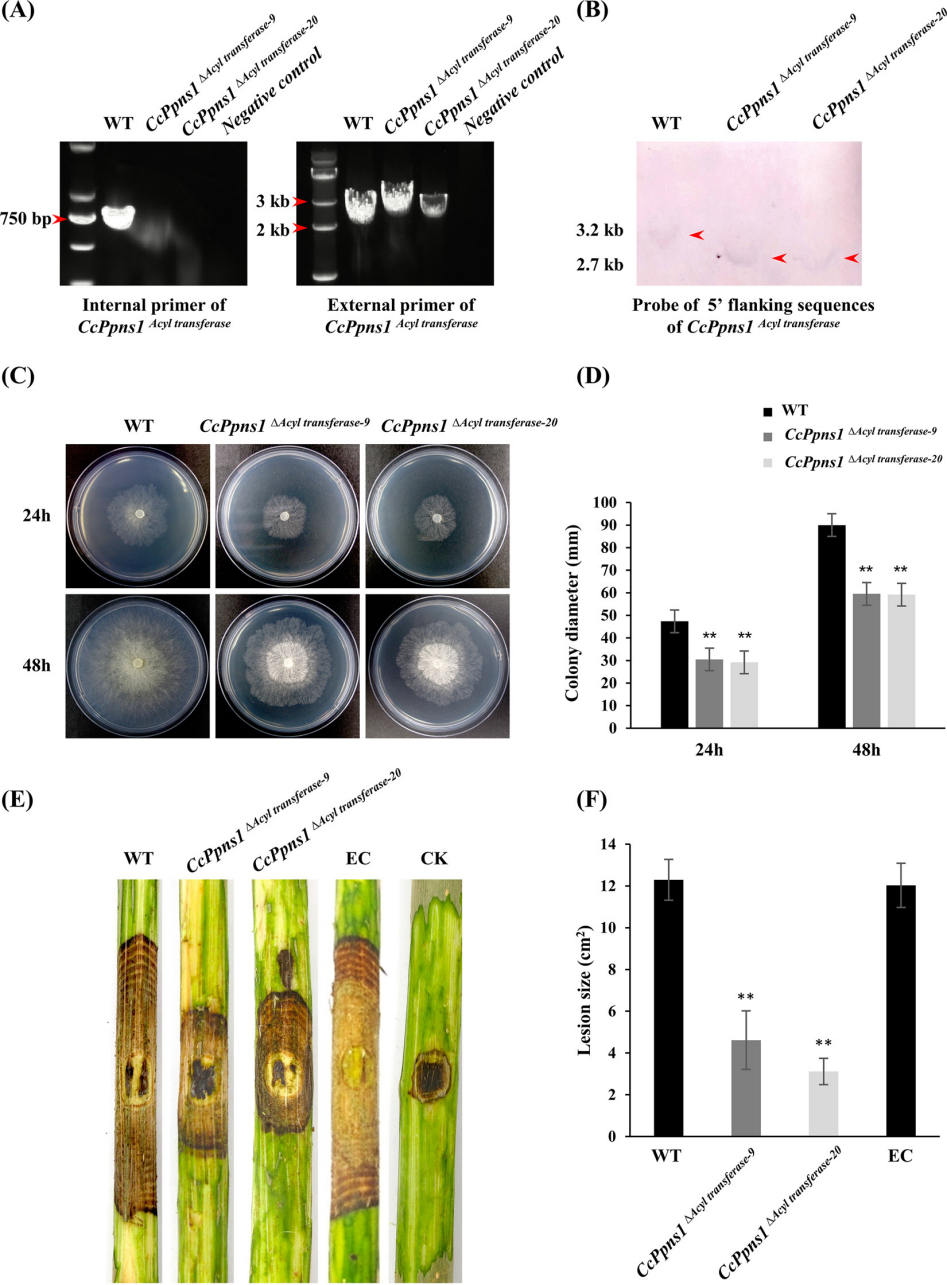
Functional characterization of the core gene of the secondary metabolism gene cluster. (A) Screening of acyl transferase domain deletion transformants by using PCR amplification with specific internal (left) and external (right) primer pairs. Negative control indicates that no template was used. (B) Southern blot analysis of genomic DNA from the wild type and two deletion mutants. Genomic DNA from these isolates was digested with SalI and probed with 5′ flanking sequences. (C) Colony morphology of the wild type and two *CcPpns1^Δacyl transferase^* mutants incubated on PDA plates. (D) Colony diameter of each strain after 1 day and 2 days of growth on PDA plates. The values show the average of three colony diameter measurements. (E) The pictures show poplar twigs inoculated with the wild type, two *CcPpns1^Δacyl transferase^* mutants, and ectopia (EC) after 7 days of incubation. CK indicates twigs inoculated with 5-mm PDA agar plugs. (F) The statistical data for lesion size on the poplar twigs inoculated with each strain. Error bars represent SD based on three independent biological replicates with three technical replicates each. The data were analyzed using one-way ANOVA followed by Duncan’s range test with SPSS 20.0. **, *P < *0.01, significant differences.

Subsequently, we analyzed the functions of the core gene *CcPpns1* of the cluster with a gene deletion method. Due to the long nucleotide sequence of *CcPpns1* (over 12 kb), a part region of this gene (the acyl transferase domain) was deleted by the split-marker method. Finally, two single-copy replacement mutants were successfully acquired according to PCR confirmation and Southern blot analysis (Fig. 8A and B). Phenotypic analyses showed that *CcPpns1^Δacyl transferase^* mutants displayed significant defects in fungal growth and virulence (Fig. 8C to F). Dramatically reduced lesion areas on poplar twigs inoculated with the *CcPpns1^Δacyl transferase^* mutant compared to the wild type and ectopic transformant were observed (Fig. 8E and F). Therefore, we speculated that the attenuated virulence of the Δ*CcPmk1* mutant may partly result from the reduced abundance of secondary metabolites derived from this gene cluster.

### Detection of metabolic differences between Δ*CcPmk1* and wild type.

**FIG 9:**
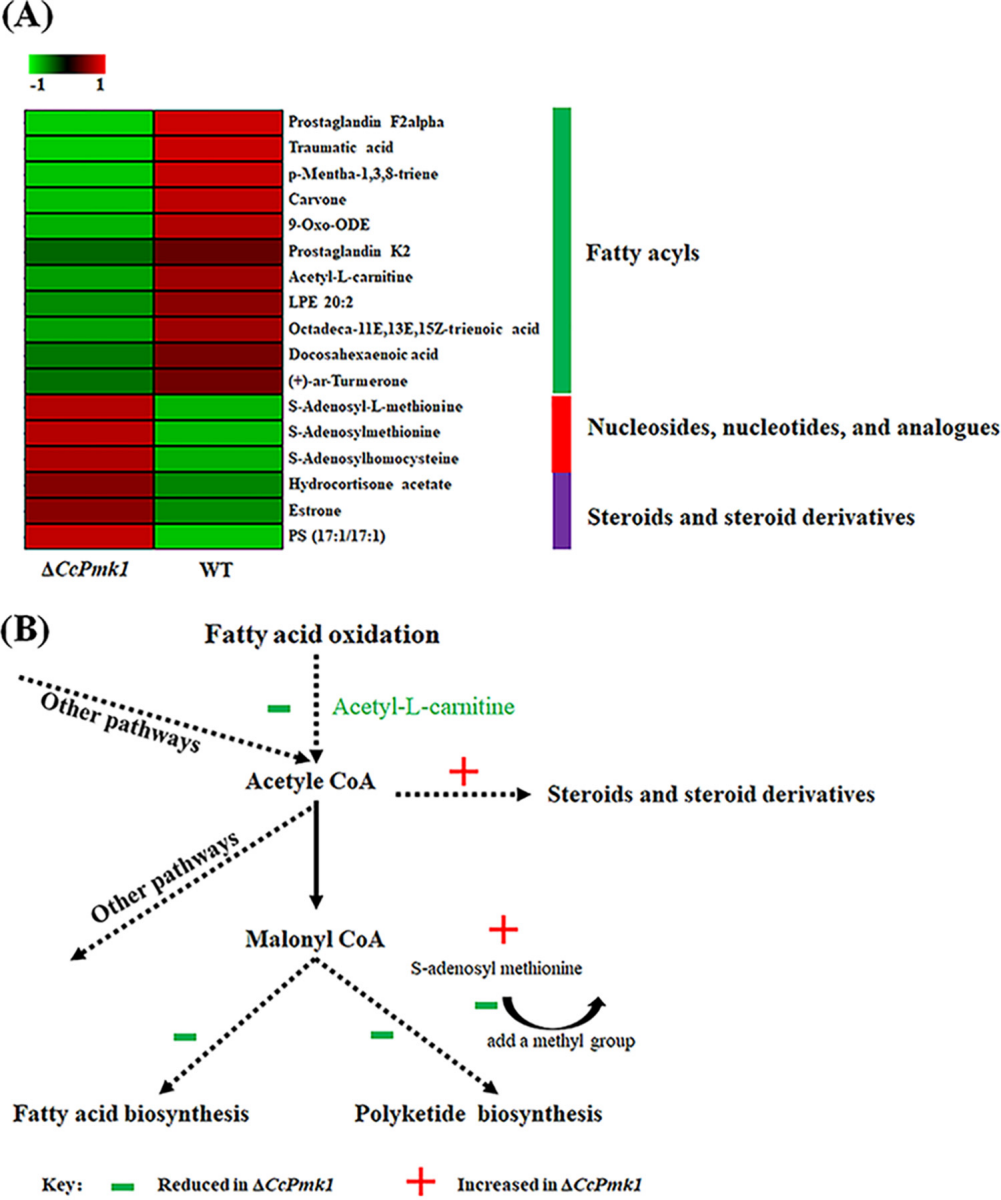
Metabolomic analysis. (A) Heatmap showing the metabolites related to lipids and lipid-like molecules and cysteine and methionine metabolism whose abundance was significantly altered. (B) Hypothesis model of metabolite production or utilization shown in panel A.

To clarify the differences in metabolites after the deletion of *CcPmk1*, 12 samples of the Δ*CcPmk1* and wild type were extracted, respectively and analyzed by mass spectrometry-based metabolomics (Fig. 1A). High Pearson correlation values between the quality control samples were obtained, indicating high data stability during LC-MS/MS analysis (Fig. S4). The metabolomics data from this experiment yielded a total of 973 metabolites in positive polarity mode. According to PCA, we found that the metabolome data for the Δ*CcPmk1* and wild type were markedly separated (Fig. S4), indicating significantly different metabolites in the Δ*CcPmk1* and wild type. Therefore, we sought to identify the altered metabolites in the Δ*CcPmk1* in greater detail and found 124 differentially regulated metabolites, including 66 significantly downregulated and 58 significantly upregulated metabolites (Table S4), of which 62 (33 downregulated and 29 upregulated) could be matched to known annotations in the KEGG, Human Metabolome Database (HMDB), or LIPID MAPS databases (Table S4). The 62 annotated metabolites are mainly involved in amino acid metabolism (17 annotations), biosynthesis of secondary metabolites (7 annotations), phenylpropanoids and polyketides (8 annotations), lipids, and lipid-like molecules (14 annotations, 2 annotations were included in biosynthesis of secondary metabolites). Importantly, most of the lipids and lipid-like molecules (11/14) were significantly downregulated in the Δ*CcPmk1* compared to the wild type, while the annotated glycerophospholipids, steroids, and steroid derivatives, putative components of cell wall, were significantly upregulated in the Δ*CcPmk1* compared to the wild type (Fig. 9A), consistent with the results of phosphoproteomic analysis (Table S4 and Fig. 2). In addition, the Δ*CcPmk1* mutant showed reduced lipid accumulation in the mycelium compared to the wild-type and complemented strains, as shown by Nile red staining (Fig. S5). KEGG enrichment analysis revealed that only cysteine and methionine metabolism was significantly enriched (Fig. S6). Further analysis revealed that three metabolites involved in this pathway accumulated at significantly greater levels in the Δ*CcPmk1* compared to the wild type, including S-adenosylhomocysteine, S-adenosyl-L-methionine, and S-adenosylmethionine (Fig. 9A).

Previous studies have showed that S-adenosyl-L-methionine can be used to provide methyl groups for the biosynthesis of polyketide metabolites. For example, methyl groups from S-adenosyl-L-methionine are added to the different chains of lovastatin through the methyltransferase domain of LNKS or lovastatin diketide synthase (LDKS) (50, 51). As shown in Fig. 9A, most of the annotated lipids and lipid-like molecules were significantly downregulated in the Δ*CcPmk1* compared to the wild type, indicating significantly reduced fatty acid biosynthesis and polyketide biosynthesis. Therefore, the reduced polyketide biosynthesis in the *CcPmk1* deletion mutant might increase the abundance of the precursor metabolites S-adenosylmethionine, S-adenosyl-L-methionine, and S-adenosylhomocysteine (which can be transformed from S-adenosylmethionine) (Fig. 9B). On the other hand, the increased precursors can then be used for the biosynthesis of other components, such as steroids and steroid derivatives, resulting in the accumulation of these metabolites in the *CcPmk1* deletion mutant (Fig. 9B).

## DISCUSSION

MAPK cascades are conserved and essential signal transduction pathways that play crucial roles in various biological processes and responses to the environmental changes through the transduction of extracellular signals in eukaryotes (3). The MAPK Pmk1, a well-characterized MAPK in plant pathogenic fungi, acts as a key regulator of fungal pathogenicity. Deletion of *Pmk1* orthologs in different phytopathogenic fungi significantly reduces or even abolishes their virulence by affecting the formation of the infection structure, invasive growth, effector or toxin secretion, and so on (3, 6, 15, 16, 19, 21). Additionally, the phosphorylation of Pmk1 by upstream Ste7 (MAP2K) is essential for its functions, and activated Pmk1 then modulates the expression of downstream transcription factors and can also direct the phosphorylation of downstream targets (52, 53). In this study, we performed a phosphoproteomic analysis of the Δ*CcPmk1* and wild type. The results showed that the abundance of phosphorylated proteins involved in intracellular hydrolase activity and PK activity was significantly reduced in the Δ*CcPmk1*, while the abundance of phosphorylated proteins involving in integral component of membrane, oxidation-reduction processes, and transmembrane transport was significantly increased in the Δ*CcPmk1*, indicating changes in metabolic strategies after *CcPmk1* deletion. Then, we assessed the metabolic differences in the Δ*CcPmk1* and wild type through untargeted metabolomics analysis. The results showed that amino acid metabolism, biosynthesis of secondary metabolites, lipids, and lipid-like molecules were significant different. Functional characterization of the downstream transcription factor CcSte12 of CcPmk1 showed that it is required for fungal growth, conidiation, stress responses, and pathogenicity. Remarkably, the results provided evidence that CcPmk1 can regulate the phosphorylation of proteins involved in secondary metabolism that may be required for fungal pathogenicity. Collectively, the identification of the regulatory network of CcPmk1 provides new clues to better understand the pathogenesis of *C. chrysosperma*.

The Pmk1 signaling pathway is a highly conserved pathogenicity-related MAPK cascade that regulates appressorium formation, penetration, or other infection processes in appressorium-forming or nonappressorium-forming pathogens (16). Remarkably, all the identified Pmk1 orthologs are essential for pathogens’ virulence until now. Pmk1 is regarded as a core direct or indirect regulator of a series of downstream targets involved in pathogenic processes. Pmk1 is also involved in the regulation of secondary metabolism in several pathogens. For example, deletion of the Pmk1 ortholog in F. graminearum and Fusarium verticillioides significantly reduced the production of deoxynivalenol and fumonisin, which are important for fungal pathogenicity (23, 24). In *C. chrysosperma*, *CcPmk1* can also regulate the expression of genes suggested to be involved in secondary metabolism, as shown by transcriptional analysis (26). Here, we were surprised to find that CcPmk1 could affect the phosphorylation of GME3434_g and GME3439_g, which are related to secondary metabolism. More importantly, the phosphorylation of GME3434_g and GME3439_g was completely abolished in the *CcPmk1* deletion mutant, and the phosphorylation of several residues in GME3436_g, CcPpns1, and GME3443_g was also completely abolished in the *CcPmk1* deletion mutant. Functional analysis of the core gene revealed that it is required for fungal virulence. Although homologs of core gene have been found in other fungal species, this gene cluster is specific to *C. chrysosperma*. These results suggest that CcPmk1 can regulate this secondary metabolism gene cluster to affect fungal virulence.

Pmk1 affects the expression of effector genes (6), which was also found in *CcPmk1* of *C. chrysosperma* (18). However, no effector candidate proteins were phosphorylated, according to phosphoproteomic analyses between the Δ*CcPmk1* and wild type (data not shown), indicating the indirect regulation of effector proteins by CcPmk1. In yeast, Kss1 or Fus3 can directly phosphorylate Ste12, resulting in the dissociation of Ste12 from the Tec1/Ste12/Dig1 complex and subsequent activation of the expression of genes involved in mating and invasive growth pathways (29). However, the phosphorylation of Ste12 does not seem to be essential for fungal pathogenicity in M. oryzae (54). Additionally, many previous studies also found that Ste12 could interact with the MADS-box transcription factor Mcm1 and then coregulate mating processes (28, 35, 36, 39). Intriguingly, only the homeodomain was found at the N-terminal region of Ste12 in yeast, while there are two tandem C_2_H_2_ zinc finger domains at the C-terminal region in addition to the N-terminal homeodomain in many phytopathogenic fungi. The homeodomain is responsible for the DNA binding of conserved sequence elements, while the functions of the C_2_H_2_ zinc finger domain have not been well characterized (28). In M. oryzae, both the homeodomain and C_2_H_2_ zinc finger domain are required for appressorium penetration and plant infection (54). Similar results were found in the Ste12 orthologs in *Colletotrichum lindemuthianum* and Cryptococcus neoformans (55, 56). In this study, we found that deletion of *CcPmk1* significantly reduced but did not abolish the phosphorylation of CcSte12, indicating that CcSte12 is also phosphorylated by kinase proteins other than CcPmk1. Consistent with this finding, similar but not identical phenotypes were observed in the *CcPmk1* deletion mutant and *CcSte12* deletion mutant. As described in other fungi, CcSte12 could also interact with CcPmk1, according to yeast two-hybrid assays. However, CcSte12 could not interact with the MADS-box transcription factor CcMcm1 in *C. chrysosperma* (Fig. S2), indicating different regulatory modules of the Ste12 ortholog in different fungi.

Ste12 is a conserved homeobox transcription factor downstream of Fus3/Kss1/Pmk1, and deletion of Ste12 orthologs in phytopathogenic fungi caused significant defects in fungal pathogenicity. For example, the virulence of the Ste12 ortholog deletion mutant was strongly attenuated in *C. parasitica* (37), *B. cinerea* (32), C. neoformans (56), and F. oxysporum (57). In M. oryzae, the *Mst12* deletion mutant was nonpathogenic to host plants but could form normal melanized appressoria, in contrast to the MoPmk1 deletion mutant. These results suggest the presence of other downstream targets of MoPmk1 in addition to Mst12, which regulates the formation of appressoria (34). Although the function of Ste12 orthologs in pathogenicity is conserved among different phytopathogenic fungi, the orthologs displayed distinct and convergent functions in other phenotypes. For example, deletion of the Ste12 ortholog in *S. macrospora* (36), F. oxysporum (30), and V. dahliae (58) did not affect vegetative growth, while the Ste12 ortholog was found to be required for fungal growth in N. crassa (38), *S. sclerotiorum* (35), and F. graminearum (39). Here, we found that CcSte12 is important for fungal growth, conidiation, pathogenicity, and the stress response, and the *CcSte12* deletion mutant shared many similar but not identical phenotypes with the *CcPmk1* deletion mutant, as found in other fungi, indicating that the Ste12 ortholog is a downstream transcription factor of the Pmk1 pathway conserved in different fungi.

In M. oryzae, MoPmk1 can also phosphorylate and weakly interact with another homeobox transcription factor, Hox7, in addition to Mst12, which acts as a direct downstream transcription factor and regulates the appressorium formation (22). Additionally, phosphorylation of Hox7 by MoPmk1 was found to be essential for appressorium development, and the phosphorylation levels of Hox7 at different serine residues were changed (22). Here, we found that CcPmk1 could also interact with CcHox7, but the phosphorylation of CcHox7 did not differ in the Δ*CcPmk1* compared to the wild type. Meanwhile, the MoPmk1-dependent phosphorylation of S158 in Hox7 was also detected in S153 of CcHox7. Therefore, we speculated that the different phosphorylation results of CcHox7 may result from the different development stages. Remarkably, we found that phosphorylation of the homeobox transcription factor GME5674_g was regulated by the CcPmk1, as it was reduced in the Δ*CcPmk1* compared to the wild type. Further analyses revealed that MGG_00184 (GME5674_g ortholog) is indispensable for conidiogenesis but not required for other processes, suggesting that this homeobox transcription factor is specifically involved in conidiation (47). In our previous study, we found that Δ*CcPmk1* could not form conidia, while the Δ*CcSte12* generated significantly fewer conidia than the wild type (18). Therefore, we speculated that CcPmk1 regulates conidiogenesis through the downstream homeobox transcription factors GME5674_g and CcSte12.

**FIG 10:**
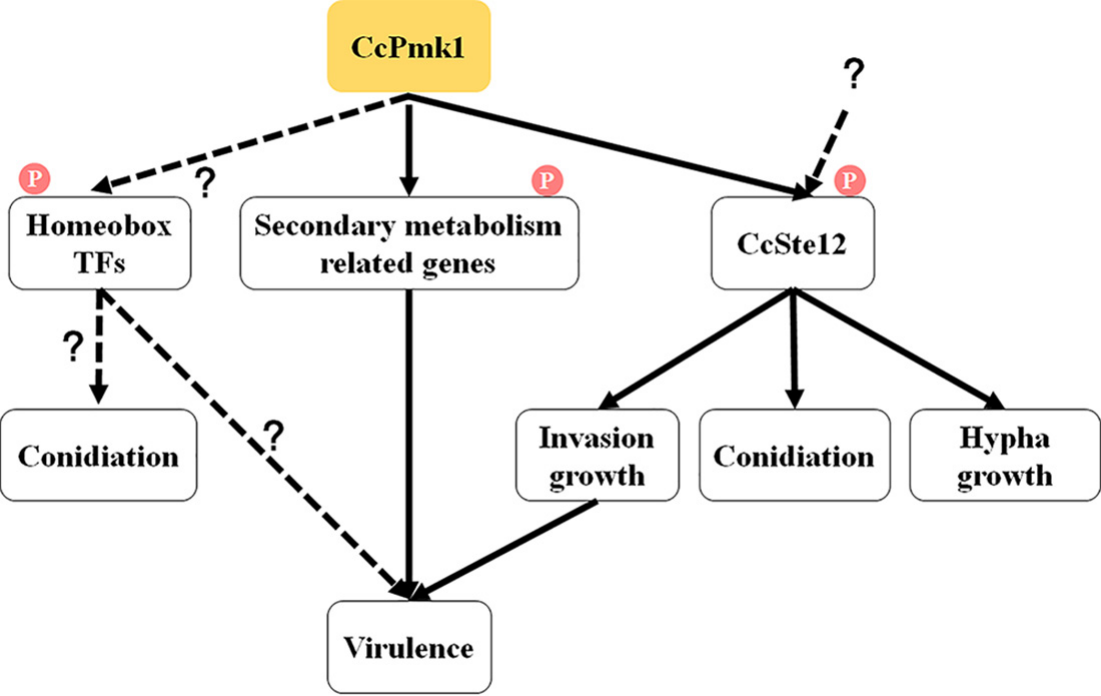
Model illustrating the hierarchical control of downstream targets associated with CcPmk1 in *C. chrysosperma*. CcSte12 is directly phosphorylated and transcribed regulated by CcPmk1. Additional pathways might also regulate CcSte12 activity. CcPmk1 can also directly phosphorylate (P) downstream proteins involved in secondary metabolism, which are important for fungal virulence. The remaining functions of CcPmk1 might be mediated by other, unknown transcriptional regulators, such as other homeobox transcription factors, in addition to CcSte12.

Collectively, these findings show that CcPmk1 acts as a core regulator of fungal pathogenicity by modulating a small number of master downstream targets, such as CcSte12, and secondary metabolism-related proteins (Fig. 10).

## MATERIALS AND METHODS

### Fungal growth conditions and pathogenicity assay.

The wild-type *C. chrysosperma* strain (CFCC 89981) and the *CcPmk1* deletion mutant were obtained and preserved in our lab. Generally, the strains were grown on potato dextrose agar (PDA) plate at 25°C or PDB medium with shaking at 150 rpm, 25°C. To calculate the growth of each strain, the wild-type, gene deletion mutant, and complemented strains were inoculated on the PDA plates and incubated at 25°C. To determine the formation of pycnidia, each strain was grown on PDA plates for 30 days. To analyze the tolerance of each strain to the H_2_O_2_, the conidial suspension (~10^6^ spores/mL) was added into the melted PDA medium and mixed well. The filter was placed on the central of the plates and supplemented with 5 μL 3 M H_2_O_2_. The inhibition zone diameter of each strain was measured. For lipid droplet staining, the fungal mycelia were harvested and stained with Nile red solution (50 mM Tris/maleate buffer, 20 mg/mL polyvinylpyrrolidone, and 2.5 μg/mL Nile red, pH 7.5) at room temperature for 5 min. For pathogenicity assay, the healthy annual poplar twigs were collected from the nursery garden, and then cut into 20-cm-long segments. The truncated twigs were scorched with a flat iron (5 mm in diameter), and inoculated with wild-type, gene deletion mutant, and complemented strains. The inoculated twigs were placed in the trays to maintain humidity and incubated at 25°C. All assays were repeated three times.

### Phosphoproteome analysis.

To prepare the phosphoproteomic samples, the wild-type and Δ*CcPmk1* strains were grown in the PDB medium supplemented with sterilized poplar branches to mimic infection status with shaking at 150 rpm and 25°C for 2 days. The cultures were filtered with Miracloth (Calbiochem) and then minced individually with liquid nitrogen and lysed in lysis buffer followed by 5 min of ultrasonication on ice. Supernatant was acquired with centrifugation at 12,000 × *g* and 4°C for 15 min. The supernatant was treated with DTT and sufficient iodoacetamide. The resulting supernatant was mixed with four times volume of precooled acetone by well vortex and incubated at -20°C for at least 2 h. The precipitation was collected by using centrifugation and washed with ice-cold acetone for twice. The pellet was dissolved by dissolution buffer (100 mM TEAB, pH 8.5 and 6 M urea). Total protein concentration was calculated by Bradford protein assay. Each strain contained three repeats.

The protein solution from each sample was digested with Trypsin Gold (Promega) at 1:50 trypsin-to-protein mass ratio at 37°C for 16 h. The peptides were desalted with C18 cartridge and vacuum-dried. The peptides were redissolved in buffer solution (250 mM acetic acid with 30% acetonitrile, pH 2.5–3.0). Modified peptides were enriched by using phos-select iron affinity gel (Sigma, P9740) according to the manufacturer’s instructions. The bounded peptides were eluted, dried and desalted using peptide desalting spin columns (Thermo Fisher, 89852).

The collected phosphopeptides were used for LC-MS/MS Analysis with an EASY-nLC 1200 ultra-high-performance liquid chromatography (UHPLC) system (ThermoFisher), coupled with an Orbitrap Q Exactive HF-X mass spectrometer (Thermo Fisher). Briefly, the phosphopeptides were dissolved in 0.1% FA and separated on a home-made analytical column (15 cm × 150 μm, 1.9 μm), using a 120-min linear gradient from 5% to 100% of eluent B (0.1% FA in 80% ACN) in eluent A (0.1% FA in H_2_O) at a flow rate of 600 nL/min. Q-Exactive HF-X mass spectrometer coupled with UHPLC. The applied electrospray voltage was 2.3 kV. The *m/z* scan range was 350–1,500 for full MS scans at a resolution of 60,000 (at 200 *m/z*). Then, the 40 most abundant precursor ions were selected for fragmentation using higher energy collisional dissociation fragment analysis at a resolution of 15,000 (at 200 *m/z*) with the dynamic exclusion parameter of 30 s. The automatic gain control (AGC) target value was set at 5e^4^.

The resulting MS/MS spectra data were searched through database by using Proteome Discoverer 2.2 (PD 2.2, Thermo). The searched parameters of a mass tolerance of 10 ppm for precursor ion scans and a mass tolerance of 0.02 Da for the product ion scans were used. A maximum of two miscleavage sites were allowed. Oxidation of methionine (M), phospho of serine (S), threonine (T), and tyrosine (Y) and acetylation of the N terminus were specified as variable modifications in PD 2.2. For protein identification, protein with at least one unique peptide was identified at false discovery rate less than 1.0% on peptide and protein level, respectively. Precursor quantification based on intensity was used for label-free quantification. The protein quantitation results were statistically analyzed by Mann-Whitney test. The differentially expressed peptides or proteins were defined as *P < *0.05 and FC >1.5 or <0.67.

### Gene ontology analysis and enrichment and pathway analysis.

GO and KEGG were used to analyze the GO category and pathway. The enrichment pipeline was used to perform the enrichment analysis of GO and KEGG, respectively (59). Significant enrichment was determined with *P < *0.05.

### Gene deletion and complementation.

Targeted gene deletion mutants were generated using the split marker method as described previously (18). Briefly, the upstream and downstream flanking sequences of target genes or regions were amplified and fused with hygromycin fragment. Then, the recombinant fragments were directly transformed into protoplasts of the wild-type strain. Transformants were selected on TB3 medium supplemented with 20 μg/mL hygromycin. The successful replacement transformants were identified by PCR assays with specific primers and confirmed by Southern blotting analysis. To complement the deleted genes, the fragment containing its native promoter, the open reading frame, and terminator regions was PCR amplified and directly co-transformed into the protoplasts of the gene deletion mutant with a Geneticin-resistant cassette. The complementary transformants were selected on TB3 medium supplemented with 20 μg/mL hygromycin and 40 μg/mL Geneticin. Successful complementary strains were screened using PCR assays with specific primers. All the primers used were listed in the Table S1.

### RNA extraction and quantitative RT-PCR analysis.

To determine the expression level of the *CcSte12* in the *CcPmk1* deletion mutant, wild-type, *CcPmk1* deletion mutant, and complemented strains were grown in the PDB medium with shaking at 150 rpm and 25°C for 2 days. The cultures were filtered with Miracloth (Calbiochem) and then flash frozen in liquid nitrogen for RNA extraction. TRIzol reagent (Invitrogen), coupled with chloroform and ethanol, were used to isolate the total RNA.

For real time quantitative reverse transcription-PCR (RT-qPCR) assays, mRNA was enriched with oligo(dT)18 primer and cDNA was synthesized using ABScript II cDNA Fist-Strand Synthesis Kit (ABclonal, China) according to the manufacturer’s instructions. The qRT-PCR assay was conducted with SuperReal Premix Plus (TIANGEN, China) with the Applied Biosystems 7,500 real-time PCR system (Applied Biosystems, USA). The *Ccactin* gene was used as an internal reference. 2^−ΔΔCt^ method was used to calculate the relative expression level.

### Yeast two hybrid.

The Matchmaker Gold Yeast Two-Hybrid System was used in this study. The full-length of *CcPmk1* and *CcSte12* was amplified with specific primers and fused to the bait construct pGBKT7. The fragments of *CcSte12*, *CcHox7*, and *CcMcm1* were amplified with specific primers and fused to the prey construct pGADT7. The resulting prey and bait constructs were cotransformed in pairs into yeast strain Y2HGold using the PEG/LiAc method. The transformants were screened on the SD-Trp-Leu and then assayed for the growth on SD-Trp-Leu-His-Ade and the expression of *MEL1* reporter gene according to the instructions.

### Bioinformatic analysis.

The domain structure was annotated with the Interpro (https://www.ebi.ac.uk/interpro/). ClustalX v.2.0 was used to sequence alignment and visualized using the BioEdit. Synteny of the gene clusters between each strain was performed by GATA (60). The gene arrangement was visualized using the IGV with gene annotation files from their genome database (https://genome.jgi.doe.gov/Cytch1/Cytch1.home.html and https://mycocosm.jgi.doe.gov/Crypa2/Crypa2.home.html).

### Untargeted metabolomics analysis.

To prepare the metabolome samples, the wild-type and Δ*CcPmk1* strains were grown in the PDB medium supplemented with sterilized poplar branches to mimic the infection status with shaking at 150 rpm and 25°C for 2 days. Each strain contained six repeats. The samples were individually collected and grounded into powder with the liquid nitrogen. The powder was resuspended with prechilled 80% methanol and 0.1% formic acid and fully mixed. The supernatant was acquired with the centrifugation at 15,000 × *g* and 4°C for 20 min. The supernatant was then diluted to final concentration containing 53% methanol by LC-MS grade water and centrifuged at 15,000 × *g*, 4°C for 20 min. The resulting supernatant was used for LC-MS/MS analysis with a Vanquish UHPLC system (ThermoFisher, Germany) coupled with an Orbitrap Q ExactiveTM HF mass spectrometer (Thermo Fisher, Germany) in Novogene Co., Ltd. (Beijing, China). Samples were injected onto a Hypesil Goldcolumn (100 × 2.1 mm, 1.9 μm) with a 17-min linear gradient at a flow rate of 0.2 mL/min. Q ExactiveTM HF mass spectrometer was operated in positive/negative polarity mode with spray voltage of 3.2 kV, capillary temperature of 320°C, sheath gas flow rate of 40 arb, and aux gas flow rate of 10 arb.

The Compound Discoverer 3.1 (CD3.1, ThermoFisher) was used to analyze the peak alignment, peak picking, and quantitation for each metabolite. The metabolites were qualitative and relative quantitative using the mzCloud (https://www.mzcloud.org/), mzVault, and MassList databases. These metabolites were annotated using the KEGG database (https://www.genome.jp/kegg/pathway.html), HMDB database (https://hmdb.ca/metabolites) and LIPIDMaps database (http://www.lipidmaps.org/). The metabolites with variable importance in projection >1 and *P* < 0.05 and FC ≥2 or FC ≤0.5 were considered to be differential metabolites.

### Statistical analysis.

Data are exhibited as mean value ± SE and SD based on three independent biological replicates with three technical replicates each. All statistical analyses were performed with one-way ANOVA followed by Duncan’s range test using SPSS 20.0. Asterisks are used to indicate significant differences, *, *P* < 0.05 and **, *P* < 0.01.

### Data availability.

The mass spectrometry proteomics data have been deposited into the ProteomeXchange Consortium via the PRIDE (61) partner repository with the data set identifier PXD032206. In addition, we have submitted our metabolomics data to MetaboLights (62) with the accession numbers: MTBLS4463 (www.ebi.ac.uk/metabolights).
