# Supplementary material for: Phosphoproteomic and Metabolomic Profiling Uncovers the Roles of CcPmk1 in the Pathogenicity of Cytospora chrysosperma
Source: Microbiol Spectr. 2022 Jun 23;10(4):e00176-22. doi: 10.1128/spectrum.00176-22 (PMC9430611; doi:10.1128/spectrum.00176-22)
Supplement: Supplemental file 2 — Supplemental material. Download spectrum.00176-22-s0002.pdf, PDF file, 1.1 MB [file spectrum.00176-22-s0002.pdf]

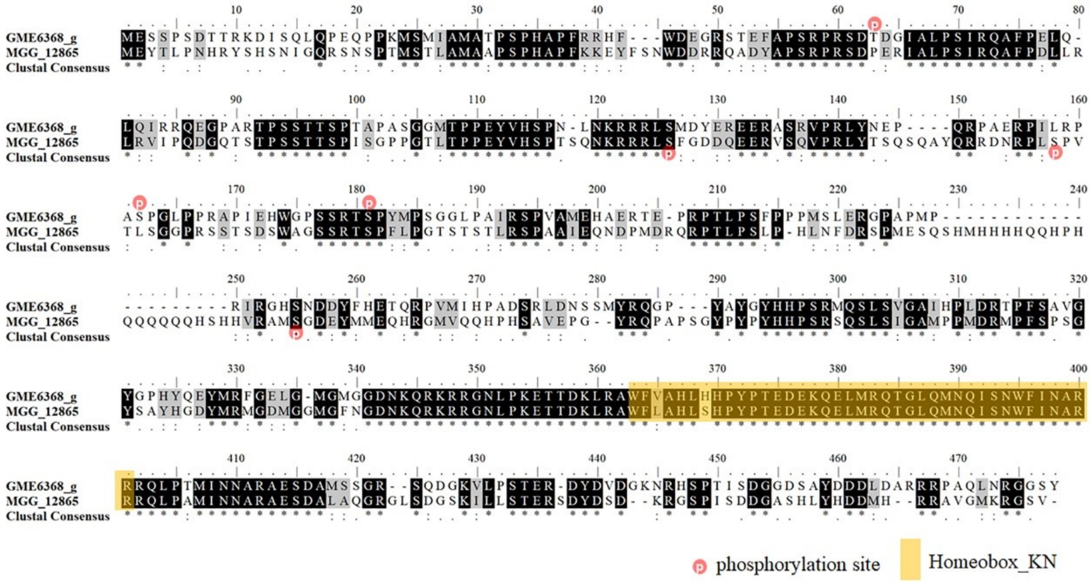

Figure S1 Amino acid sequence alignment of CcHox7 and MoHox7. The yellow box represents the Homeobox_KN domain. The pink circles indicate the putative phosphorylated sites. Absolutely conserved residues were shaded black.

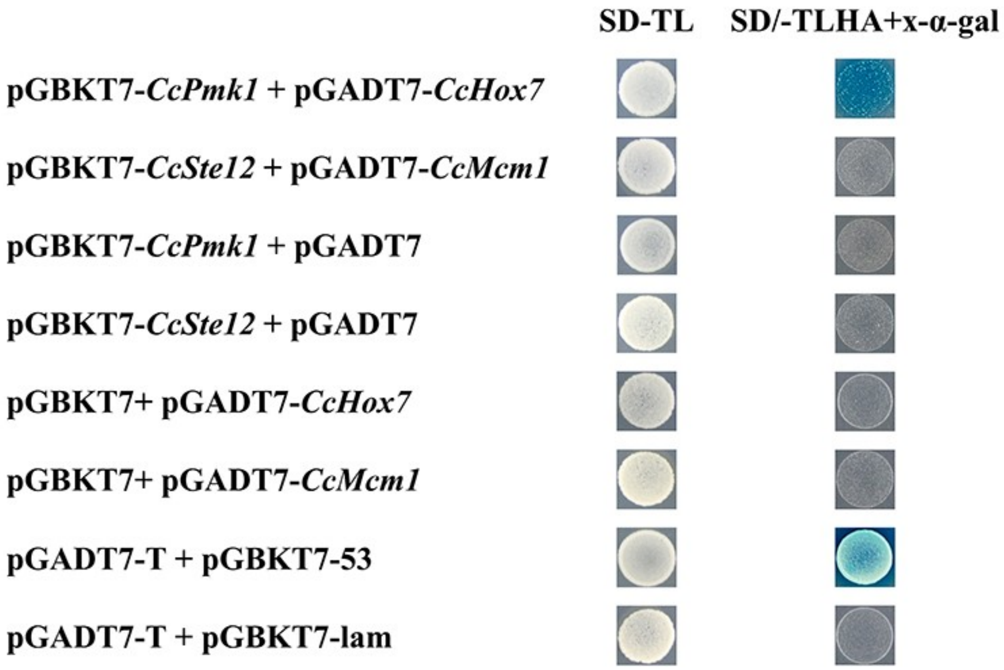

Figure S2 Yeast two-hybrid assay of CcPmk1 and CcMcm1, CcPmk1 and CcHox7, and CcSte12 and CcHox7. SD-TL represents SD medium lacing Trp and Leu. SD-TLHA represents SD medium lacing Trp, Leu, His, and Ade.

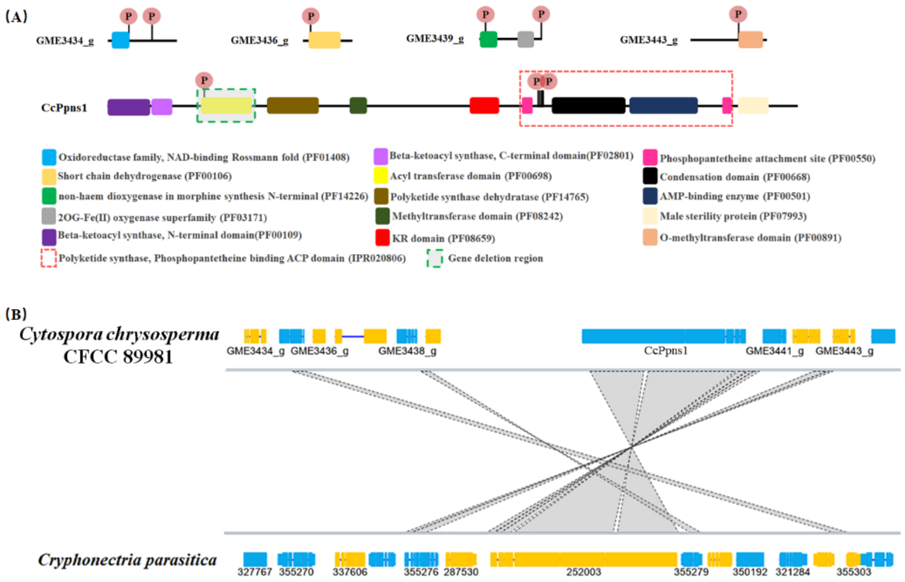

Figure S3 Domain analysis and sequence alignment of secondary metabolism genes. (A) Domain structure of phosphoproteins and phosphosites of protein involved in putative secondary metabolism. Different color boxes indicate different domain categories. The grey box with green dotted line represents the recombination region. (B) Global view of syntenic alignments of secondary metabolism gene cluster between the *Cytospora chrysosperma* and *Cryphonectria parasitica*. The syntenic alignment was analyzed by TBtools.

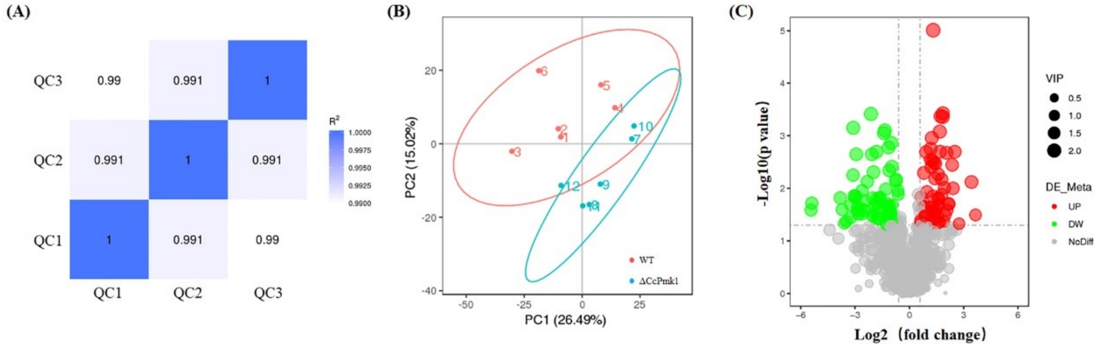

Figure S4 Analysis the metabolome data between ΔCcPmk1 and WT. (A) Pearson correlation of three quality control (QC) samples during the LC-MS/MS analysis. (B) Principal component analysis of the Δ*CcPmk1* and WT metabolic samples. (C) The volcano diagram showed the significantly expressed metabolites in Δ*CcPmk1* compared to WT. The significantly changed metabolites were defined as VIP > 1, *p* value < 0.05 and [fold change, FC] ≥ 2 or ≤ 0.5.

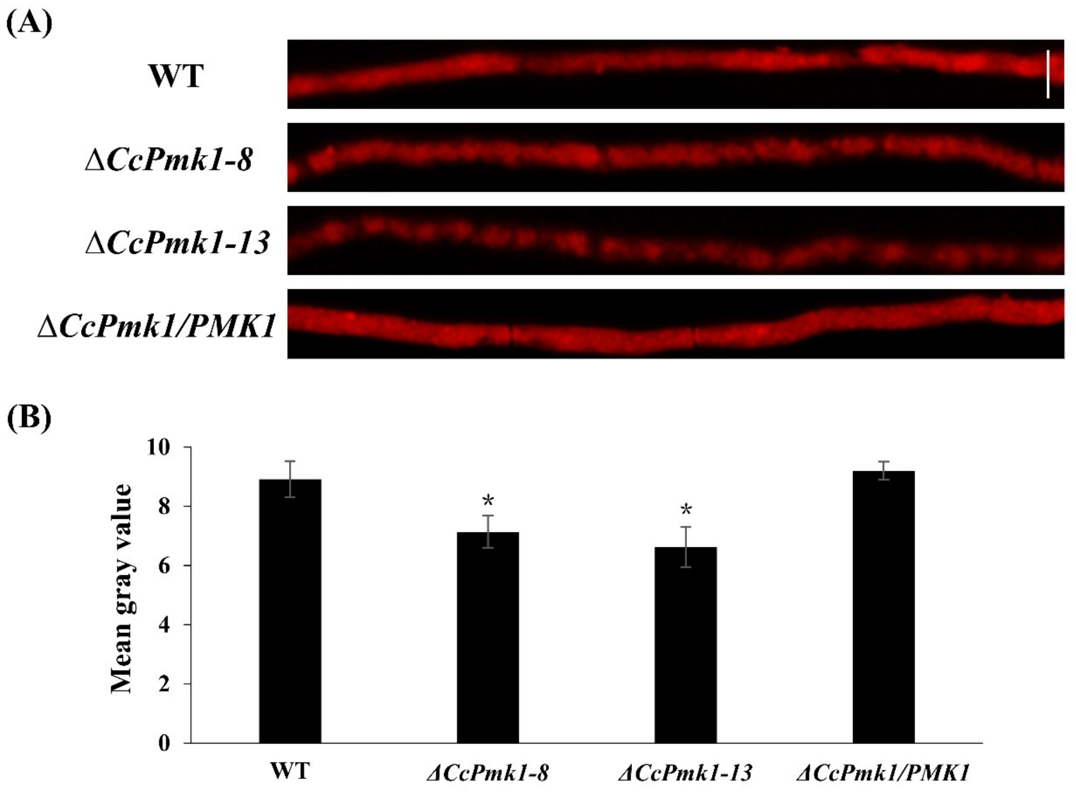

Figure S5 Lipids in mycelium of wild type, Δ*CcPmk1* and the complemented strains. (A) Hyphae were stained with Nile red and observed at 5 mins. Bar = 50 μm. (B) Mean gray value of stained hyphae were quantified by ImageJ. The error bars represent the standard deviations based on three independent biological replicates with three technical replicates each. The data were analyzed using one-way ANOVA followed by Duncan's range test with SPSS 20.0. The asterisks indicate significant differences ($P < 0.05$).

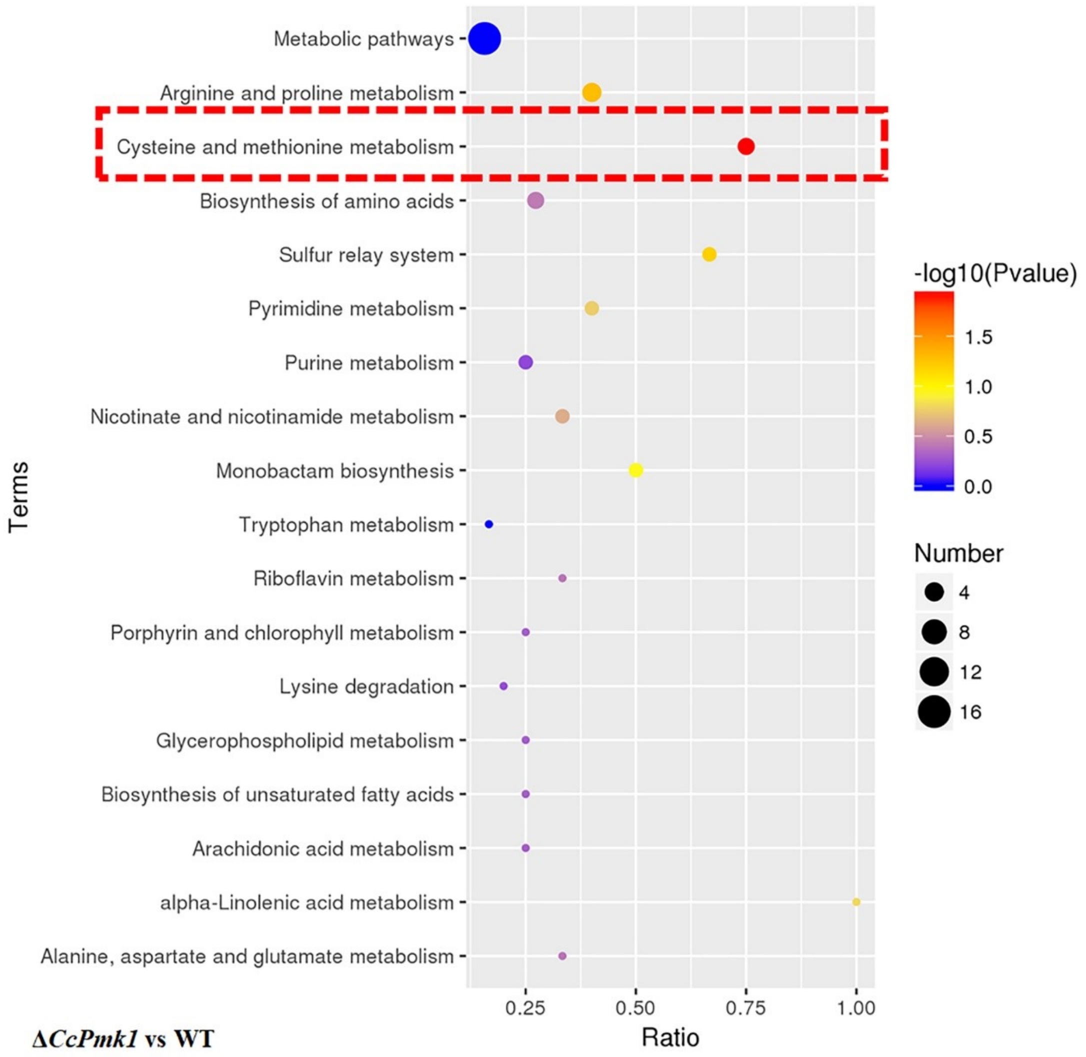

Figure S6 The scatterplot showed the enriched KEGG pathway of differentially changed metabolites. The cysteine and methionine metabolism pathway was significantly enriched at p value < 0.05.

Table S1 All primers used in this study.

Primer name	Sequence	Use in this study
**CcPmk1for**	ATGTCTCGAGCCCAGCCCCC	Sequence of *CcPmk1*
**CcPmk1rev**	CCGCATGATTTCCTGGTAGA	
**CcSte12for**	ATGTATTCCCAAGCTGGCGC	Sequence of *CcSte12*
**CcSte12rev**	CATCATGACGGCGGTGTGGG	
**TTCcSte12for**	GCCTGATTACAACCGAGTCC	Transcripts test of *CcSte12*
**TTCcSte12rev**	CGTTTGCATGCTGTTGAAGT	
**qRTCcSte12for**	AGCCAACTCCTCGTCTTTCA	qRT-PCR of *CcSte12*
**qRTCcSte12rev**	CATCTGAGAAGCCATCGACA	
**CcSte12-5Ffor**	TCACTTGGACCTGTGCTCTG	5F flanking sequence of *CcSte12*
**CcSte12-5Frev**	CCCAAGACGTGACCAAAACT	
**CcSte12-3Ffor**	AAAGCCATACGCACAAAACC	3F flanking sequence of *CcSte12*
**CcSte12-3Frev**	CAGCTCCGAGACAAACAACA	
**CcSte12-5Foverlap**	gtcgtgactgggaaaaccctggcgCCCAAGACGTGACCAAAACT	5F flanking sequence of *CcSte12* combinate 2/3rd sequence of the hygromycin cassette
**CcSte12-3Foverlap**	tcctgtgtgaaattgttatccgctAAAGCCATACGCACAAAACC	3F flanking sequence of *CcSte12* combinate 2/3rd sequence of the hygromycin cassette
**HY-R**	GTATTGACCGATTCCTTGCGGTCCGAA	The 2/3rd portion of the hygromycin cassette
**YG-F**	GATGTAGGAGGGCGTGGATATGTCCT	
**CcActinfor**	GATCTGGCACCACACCTTCT	*CcActin* sequence used for validation of *CcSte12* mutant
**CcActinrev**	GCTCTCGTCGTACTCCTGCT	
**Internal-CcSte12for**	CACCCTCTTTCGTCTCTTCG	Internal sequence used for validation of *CcSte12* mutant
**Internal-CcSte12rev**	TTCTGTGGGTAGGGTCCAAG	

**Hygromycinfor**	CGCCAGGGTTTTCCCAGTCACGAC	Hygromycin cassette
**Hygromycinrev**	AGCGGATAACAATTTCACACAGGA	
**ProbeHPHfor**	agccccacttgtagcagtag	Probe HPH sequence used for hybridization
**ProbeHPHrev**	ccccaatgtcaagcacttcc	
**ProbeCcSte12for**	CACCCTCTTTCGTCTCTTCG	Probe sequence of *CcSte12* used for hybridization
**ProbeCcSte12rev**	CTGCTCATCGTTGTGCATCT	
**CcSte12-Compfor**	CTCTGGTACTTGGACTGTGC	*CcSte12* complementary sequence
**CcSte12-Comprev**	CGTGGGAAATCCGTTGTAGG	
**G418-for**	GACGTTAACTGATATTGAAGGA	Geneticin-resistant cassette
**G418-rev**	GCTGGTGACGGAATTTTCAT	
**CcMcm1-for**	ATGGCCGACATCACAGATCA	Sequence of *CcMcm1*
**CcMcm1-rev**	TGACTGATGCTGGGCGTGCT	
**CcHox7-for**	ATGTCGATGATTGCTATGGC	Sequence of *CcHox7*
**CcHox7-rev**	GTAGCTGCCGCCACGGTTCA	
**AT-5Ffor**	GTGGAAGCTGCTTGAGAACC	5F flanking sequence of *CcPpns1*$^{acyl\ transferase}$
**AT-5Frev**	GTGAGTGGAAGAGGGATGGA	
**AT-3Ffor**	GATGACGTCGAGGCTTTCTC	3F flanking sequence of *CcPpns1*$^{acyl\ transferase}$
**AT-3Frev**	TGCTCGACAAACTGTCCAAG	
**AT-5Foverlap**	gtcgtgactgggaaaaccctggcgGTGAGTGGAAGAGGGATGGA	5F flanking sequence of *CcPpns1*$^{gacyl\ transferase}$ combinate 2/3rd sequence of the hygromycin cassette
**AT-3Foverlap**	tcctgtgtgaaattgttatccgctGATGACGTCGAGGCTTTCTC	3F flanking sequence of *CcPpns1*$^{gacyl\ transferase}$ combinate 2/3rd sequence of the hygromycin cassette
**External-ATfor**	ACGCTCGGTGAGTACCAAGT	External sequence used for validation of *CcPpns1*$^{\Delta acyl\ transferase}$
**External-ATrev**	GGATCGACTTCAGCCATGTT	

**Internal-ATfor**	AGCAGAGGGTCCAGCATCTA	Internal sequence used for validation of *CcPpns1*$^{\Delta acyl\ transferase}$
**Internal-ATrev**	GTCCAACCTCAAGAGCGAAG	
**ProbeATfor**	ATGGGCCAAGCATGACTATC	Probe 5' flanking sequence of *CcPpns1*$^{acyl\ transferase}$ used for hybridization
**ProbeATrev**	CTGATACACCGGCTCAACCT	
**qRTCcActinfor**	TCGGTATGGGTCAGAAGGAC	qRT-PCR of CcActin
**qRTCcActinrev**	GGAGCCTCAGTCAACAGGAC	

Table S2 Significantly changed phosphorylated protein kinases and their orthologs.

**Gene ID**	**Yeast orthologs**	***Fusarium graminearum* orthologs**	**Mutant's phenotypes in *Fusarium graminearum***	**Reference**
**GME5415_g**	RAD53	FGSG_00433	unaffected_pathogenicity	Wang et al., 2011
**GME2089_g**	STE7	FGSG_09903	reduced fungal growth, conidiation and virulence	Wang et al., 2011
**GME6481_g**	MKK1	FGSG_07295	reduced fungal growth, conidiation and virulence	Wang et al., 2011
**GME7417_g**	-	FGSG_02152	Not identified	
**GME9212_g**	FUS3	FGSG_06385	reduced fungal growth, conidiation and virulence	Wang et al., 2011
**GME7571_g**	KIN3	FGSG_09408	Mutant not acquired	Wang et al., 2011
**GME5754_g**	-	FGSG_00362	reduced fungal growth, loss of conidiation and virulence	Wang et al., 2011
**GME1117_g**	PKP1	FGSG_01963	unaffected_pathogenicity	Wang et al., 2011
**GME6695_g**	PHO85	FGSG_05393	Mutant not acquired	Wang et al., 2011

significant down-regulated

significant up-regulated

Green gene ID represents significant down-regulated

Red gene ID represents significant up-regulated

Table S3 Phosphorylation analysis of homeobox or homeodomain transcription factors in *Cytospora chrysosperma*.

Gene ID	Phosphorylation event	Peptide Sequence	Peptide abundance changes(Δ*CcPmk*1 vs WT)	Protein Abundance changes(Δ*CcPmk*1 vs WT)
GME6465_g (Ste12 ortholog)	Yes	DMFEQLSR	down	down
		HASMPAYGLEYSPAPSFVSSHYEDYSNR	none	
		SHSCPIPTCGR	none	
		RRSSIPPGIAAITAATGAAAGQAHHR	none	
		SATVMELGPYPQK	down	
		GYGTNNVYSVIEGSPTYK	down	
GME5674_g	Yes	LDSPPQGDIFPWSR	none	down
		AASANGPLSGK	none	
		SPDQQHPMWK	none	
		ASSLASTSSTFR	down	
GME3965_g	Yes	TQTPSPATPGSHNAGSSSK (phosphorylated sites not sure)	none	none
GME4027_g	Yes	KRSFTIPHGGPAP	none	none
		SRPDNLQSPLR	none	
GME5631_g	Yes	SASVGYFANR	down	none
		REETPVEPEEPRK	none	
		DHASPPSTSAETLR	none	
GME6368_g	Yes	TSPYMPSGGLPAIR	none	none

(Hox7 ortholog)		SDTDGIALPSIR	none	
		LYNEPQRPAERPILRPASPGLPPR	none	
GME754_g	No	-	-	-

49
